# Flexibility and Preorganization of Fluorescent Nucleobase-Pyrene Conjugates Control DNA and RNA Recognition

**DOI:** 10.3390/molecules25092188

**Published:** 2020-05-07

**Authors:** Željka Ban, Josipa Matić, Biserka Žinić, Anders Foller Füchtbauer, L. Marcus Wilhelmsson, Ivo Piantanida

**Affiliations:** 1 Division of Organic Chemistry and Biochemistry, Ruđer Bošković Institute, Bijenička Cesta 54, Zagreb 10000, Croatia; Zeljka.Ban@irb.hr (Ž.B.); josipa.matic@irb.hr (J.M.); Biserka.Zinic@irb.hr (B.Ž.); 2 Department of Chemistry and Chemical Engineering/Chemistry and Biochemistry, Chalmers University of Technology, S-41296 Gothenburg, Sweden; foller@chalmers.se

**Keywords:** DNA/RNA recognition, hydrogen bonding, fluorescent nucleobase, pyrene, intramolecular pre-organization

## Abstract

We synthesized a new amino acid-fluorescent nucleobase derivative (qAN1-AA) and from it two new fluorescent nucleobase–fluorophore (pyrene) conjugates, whereby only the analogue with the longer and more flexible linker (qAN1-pyr2) self-folded into intramolecularly stacked qAN1/pyrene conformation, yielding characteristic, 100 nm-red-shifted emission (λ_max_ = 500 nm). On the contrary, the shorter and more rigid linker resulted in non-stacked conformation (qAN1-pyr1), characterized by the emission of free pyrene at λ_max_ = 400 nm. Both fluorescent nucleobase–fluorophore (pyrene) conjugates strongly interacted with ds-DNA/RNA grooves with similar affinity but opposite fluorescence response (due to pre-organization), whereas the amino acid-fluorescent base derivative (qAN1-AA) was inactive. However, only intramolecularly self-folded qAN1-pyr2 showed strong fluorescence selectivity toward poly U (Watson–Crick complementary to qAN1 nucleobase) and poly A (reverse Hoogsteen complementary to qAN1 nucleobase), while an opposite emission change was observed for non-complementary poly G and poly C. Non-folded analogue (qAN1-pyr1) showed no ss-RNA selectivity, demonstrating the importance of nucleobase-fluorophore pre-organization.

## 1. Introduction

Over the past decades, fluorescent base analogues have received significantly increasing attention [1,2,3,4,5]. They can replace the natural bases in nucleic acids, and give them emissive properties that are often highly responsive to their immediate surroundings, and they have been used to monitor local environment or environmental changes upon nucleic acid conformational rearrangements [2,4,6,7,8,9]. A few, on the other hand, have emissive properties that are virtually insensitive or have a low responsiveness to their local environment and hence, for example, are useful as bright nucleic acid labels or as donor and acceptor of FRET-pairs [1,5,10,11,12]. One popular strategy to improve the emissive properties of fluorescent base analogues is to increase conjugation by extending the aromatic ring system of their natural counterparts. This often results in increased aromatic stacking-interactions with the neighboring nucleobases, which in turn is sometimes needed to compensate for a slightly decreased hydrogen-bonding ability. Recently, a family of quadracyclic fluorescent adenine analogues was developed [13,14,15], of which the qAN-family, derivatives with a nitrogen atom replacing one of the carbons in the outer ring [14], turned out to be highly fluorescent. These four-ring-system adenine analogues preserve the hydrogen-bonding capacity but contain two additional ring systems that are facing the major groove of B-form DNA. The most promising member of these derivatives, qAN1, has been reported to be an excellent fluorescent base analogue inside DNA as well as a base-base FRET-donor [16]. qAN1 is a typical example of an enlarged aromatic system that compared to its natural counterpart, adenine, has an increased aromatic stacking-interaction inside base-stacks, giving rise to increased average melting temperature of duplexes containing qAN1 in place of A [16]. With the previously reported increased aromatic stacking and promising emissive properties we hypothesized that qAN1 could be an interesting DNA-intercalator in itself or as part of homo- or heterodimer intercalator complexes that could report on DNA binding events via a fluorescence response.

Pyrene derivatives are sensitive fluorescent probes widely used for the characterization of micro-heterogeneous systems [17,18,19] because of their long emission lifetime (˃100 ns) and the ability to form excimers, as well as their pronounced hydrophobicity. Moreover, pyrene can only form a limited number of typical interactions with DNA/RNA: Aromatic stacking intercalation into DNA/RNA, binding within the DNA minor groove via a combination of hydrophobic and edge-to-face aromatic interactions, or by forming pyrene excimer within the DNA minor groove or RNA major groove [20]. Pyrene is also prone to form exciplex in combination with other chromophores. For instance, we recently reported the formation of a pyrene–quinoline exciplex with an emission maximum that was 60 nm bathochromically shifted compared to the common pyrene emission [21].

As a result of these recent finding, we decided to design hybrid compounds that combine two fluorophores; the fluorescent nucleobase analogue qAN1 and pyrene. Although both fluorophores are characterized by similar excitation and emission wavelength regimes, they significantly differ in hydrophobicity and in particular only qAN1 has the ability to form H-bonds with the target biomolecule (DNA or RNA).

**Scheme 1:**
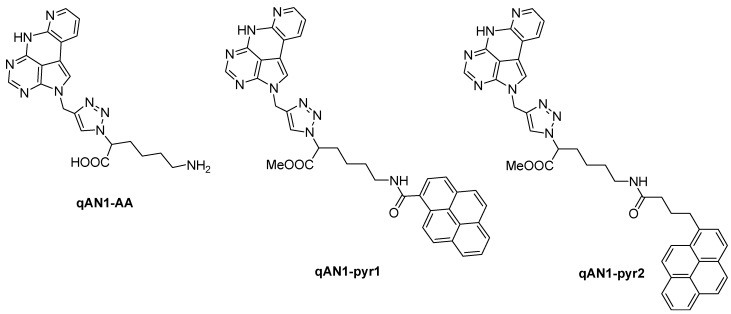
Novel qAN1-amino acid **qAN1-AA** and pyrene conjugates **qAN1-pyr1** (shorter, more rigid linker) and **qAN1-pyr2** (longer, more flexible linker).

In the design of a linker connecting the two fluorophores we opted for the preparation of a new amino acid analogue of qAN1 including a triazole unit (**qAN1-AA**, Scheme 1), coupling it with either a short and rigid pyrene analogue (**qAN1-pyr1**) or a longer and more flexible one (**qAN1-pyr2**). We expected the difference in linker length and flexibility to have an influence on the aromatic stacking interactions between the two large aromatic systems, pyrene and qAN1. Such aromatic interactions could result in intra- or inter-molecular stacked structures, formed either spontaneously in aqueous solution or upon binding to the particular DNA or RNA target of interest. Both types of interactions events could eventually result in a highly selective fluorimetric response to the DNA/RNA target.

## 2. Results and Discussion

### 2.1. Chemistry

In this work, we report the development of a synthetic strategy towards multifunctional ligands for DNA and RNA, which contain the adenine analogue qAN1 linked to amino acid conjugates. Among various possibilities to attach the qAN1 to an amino acid side chain, we opted for a copper-catalyzed alkyne-azide cycloaddition (CuAAC) approach, since this “click” chemistry approach is well established for the preparation of 1,4-disubstituted-1,2,3-triazoles [22,23,24,25,26,27,28,29,30,31,32,33,34,35,36]. This approach would allow qAN1 to be attached to the amino acid side-chain prior to or after peptide construction, depending on synthetic strategy. Furthermore, triazole units are often able to interact favorably with biological targets (DNA/RNA, proteins) through hydrogen bonding, dipole-dipole and π–π stacking interactions [37,38,39,40].

Our synthetic approach included the preparation of a novel propargylated qAN1, and its application with an amino acid azido derivative, which, by the assistance of CuAAC “click” chemistry, provided the desired triazole-linked qAN1-amino acid conjugate.

**Scheme 2:**
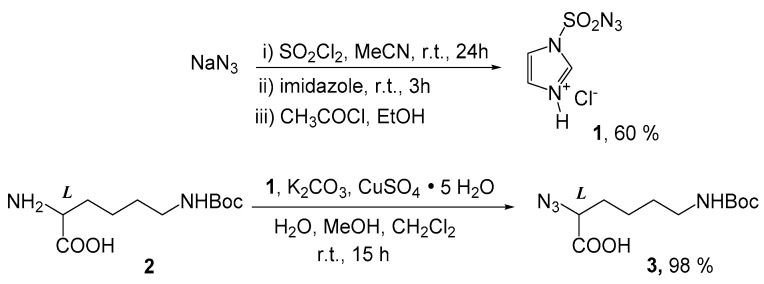
Synthesis of azido-amino acid N_3_-Lys(Boc)-OH (**3**).

The conversion of H-Lys(Boc)-OH (**2**) to azide N_3_-Lys(Boc)-OH (**3**) was performed using the freshly prepared diazotransfer reagent imidazole-1-sulfonyl azide hydrochloride (**1**) [41] (Scheme 2).

**Scheme 3:**
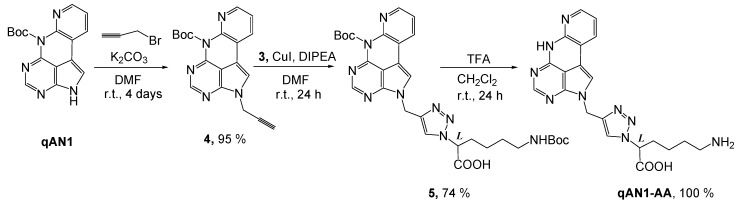
Synthesis of qAN1-amino acid conjugate **qAN1-AA**.

The reaction of **qAN1** with propargyl bromide in DMF with K_2_CO_3_ gave propargylated qAN1 (**4**) in 95% yield (Scheme 3). Alkyne **4** was coupled to azide **3** using standard CuAAC conditions in anhydrous DMF and DIPEA at room temperature overnight, resulting in conjugate **5** in good yield. Compound **5** was deprotected with TFA/CH_2_Cl_2_ to afford the target triazole linked qAN1-amino acid conjugate **qAN1-AA** in quantitative yield.

**Scheme 4:**
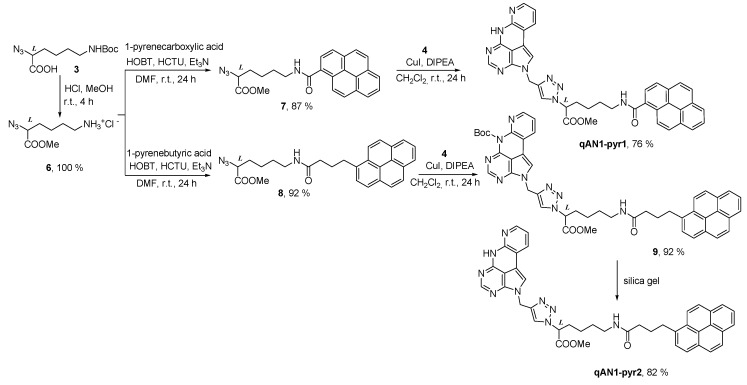
Synthesis of qAN1-amino acid-pyrene conjugates **qAN1-pyr1** and **qAN1-pyr2**.

The same “click” protocol was used for the synthesis of the qAN1-amino acid-pyrene conjugates qAN1-pyr1 and qAN1-pyr2 (Scheme 4).

For this purpose, compound **3** was treated with HCl/methanol to remove the acid-labile Boc protecting group and simultaneously esterify the carboxylic acid, affording the methyl ester **6** in quantitative yield. Using standard HCTU/HOBt coupling conditions with triethylamine in DMF [42] the derivative **6** was reacted with 1-pyrenecarboxylic or 1-pyrenebutyric acid, to give azido-pyrene ligands **7** (87%) and **8** (92%), respectively.

The protected qAN1-amino acid-pyrene conjugate **9** was readily synthesized from propargylated qAN1 **4** and azido ligand **8** through CuAAC “click” conditions employing CuI and DIPEA in dichloromethane. Further evaporation of the solvent and the addition of MeOH gave the protected product **9** as a yellow powder in 92% yield. Interestingly, when compound **4** was coupled to the azido ligand **7** under identical conditions as for the preparation of compound **9**, the Boc group spontaneously cleaved on the silica gel plates during elution, yielding a final conjugate **qAN1-pyr1** in 76% yield. This procedure also proved useful in a Boc deprotection of pyrene conjugate **9**, whereby instead of deprotection with TFA/CH_2_Cl_2_, compound **9** was spontaneously deprotected during elution on the silica gel plate. Using CH_2_Cl_2_/MeOH (20:1) as eluent, deprotected conjugate **qAN1-pyr2** was isolated in 82% yield.

### 2.2. Characterization of qAN1-Derivatives in Aqueous Solutions

Upon examination of water solubility, we found the three final compounds (**qAN1-AA**, **qAN1-pyr1**, **qAN1-pyr2**) to be moderately soluble in water, and that their aqueous solutions were stable at least for a day at a room temperature, which was checked by excellent reproducibility of the UV/Vis and fluorimetric calibration experiments (Suppl. Info. Figures S1–S3) several times during the day. For easier handling in the experiments in this study, 5 mM stock solutions of the three compounds were prepared in DMSO. These stock solutions were further diluted in an aqueous buffer immediately prior to use. The total DMSO content in the experiments was kept below 0.2%.

**Figure 1:**
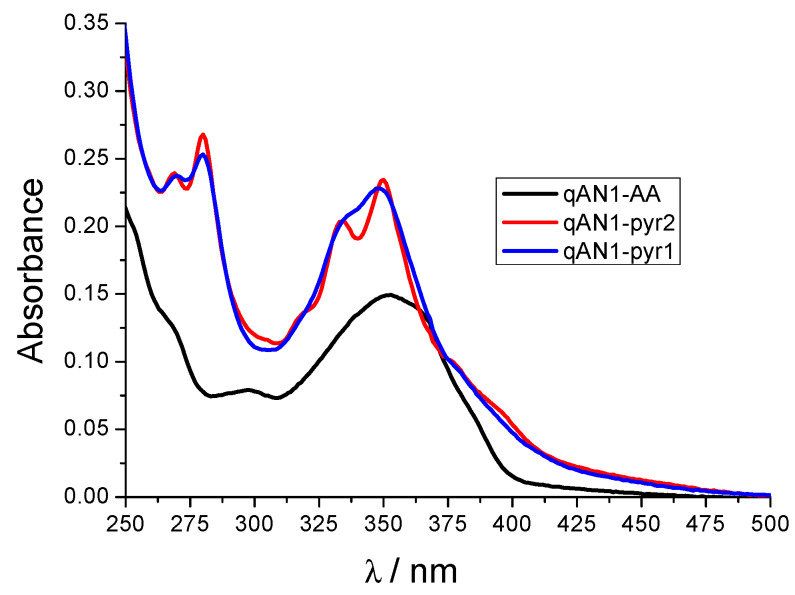
UV/Vis spectra of **qAN1-AA** and pyrene conjugates **qAN1-pyr1** and **qAN1-pyr2** (*c* = 2 × 10^−5^ M) in sodium cacodylate buffer (pH 7.0, *I* = 0.05 M) at room temperature.

The absorbance (UV/Vis spectra) of the studied compounds was proportional to their concentrations up to at least *c* = 20 µM (Suppl. Info. Figure S1–S3). The shape of the **qAN1-AA** UV/Vis spectrum (Suppl. Info. Figure S1) agreed well with data reported for **qAN1** [14] and significantly differed from UV/Vis spectra of pyrene conjugates **qAN1-pyr1** (Suppl. Info. Figure S2) and **qAN1-pyr2** (Suppl. Info. Figure S3) due to the pyrene absorbance contribution (Figure 1). The UV/Vis spectra of **qAN1-pyr1** and **qAN1-pyr2** were similar but the changes induced by heating the aqueous solutions were significantly different (Suppl. Info. Figure S1–S3). The UV/Vis spectra of **qAN1-AA** and **qAN1-pyr1** displayed no significant changes upon heating to 90 °C, whereas the corresponding spectrum of **qAN1-pyr2** at temperatures > 40 °C strongly broadened and the baseline > 500 nm significantly raised above zero, indicating scattering effects (Suppl. Info. Figure S3C). Upon reaching 95 °C, the baseline of the UV/Vis spectrum in aqueous solution of **qAN1-pyr2** returned to zero, indicating the dissolution of all colloids. Importantly, the UV/Vis spectrum of **qAN1-pyr2** aqueous solution at 95 °C closely resembled to the corresponding spectrum in methanol (Suppl. Info. Figure S3), but with a bathochromic shift (Δλ ≈ +8 nm) and hypochromic effect for the former sample (Suppl. Info. Figure S3). These findings suggest a strong aromatic stacking of **qAN1-pyr2** in aqueous solution at the room temperature, which is lost upon heating or changing the solvent to methanol.

**Figure 2:**
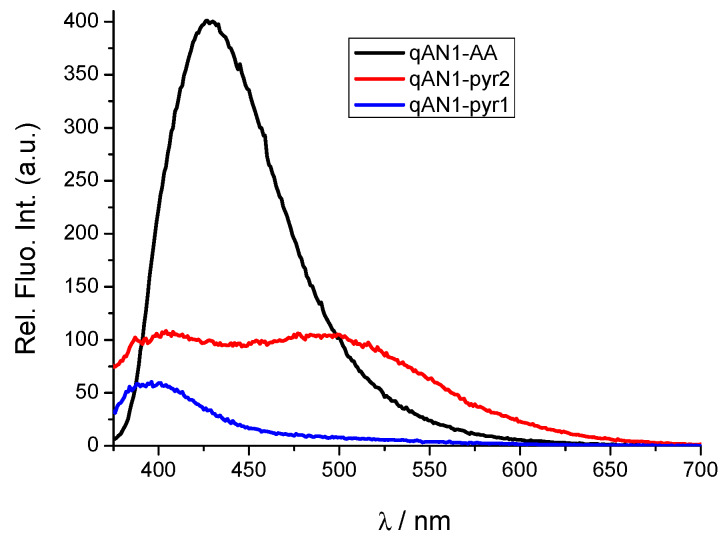
Fluorescence spectra of **qAN1-AA** and pyrene conjugates **qAN1-pyr1** and **qAN1-pyr2** (λ_exc_ = 350 nm, *c* = 1 µM) in sodium cacodylate buffer (pH 7.0, *I* = 0.05 M).

Similar to the absorption, the fluorescence of **qAN1-AA** (Figure 2) closely resembled previously reported data for **qAN1** [14], which suggests that the attachment of a triazole-amino acid tail does not influence absorption nor emission of the qAN1-chromophore.

**Figure 3:**
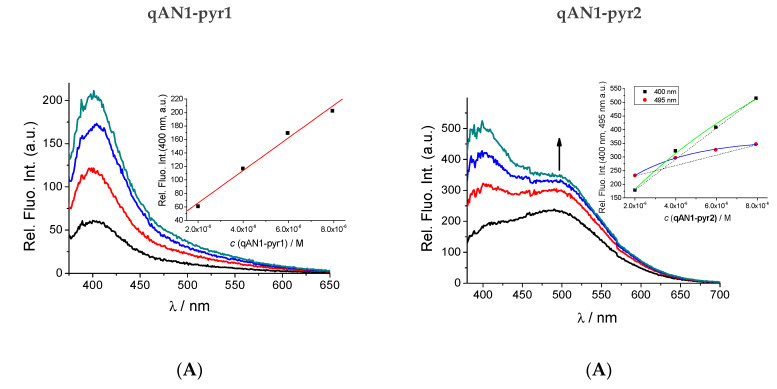

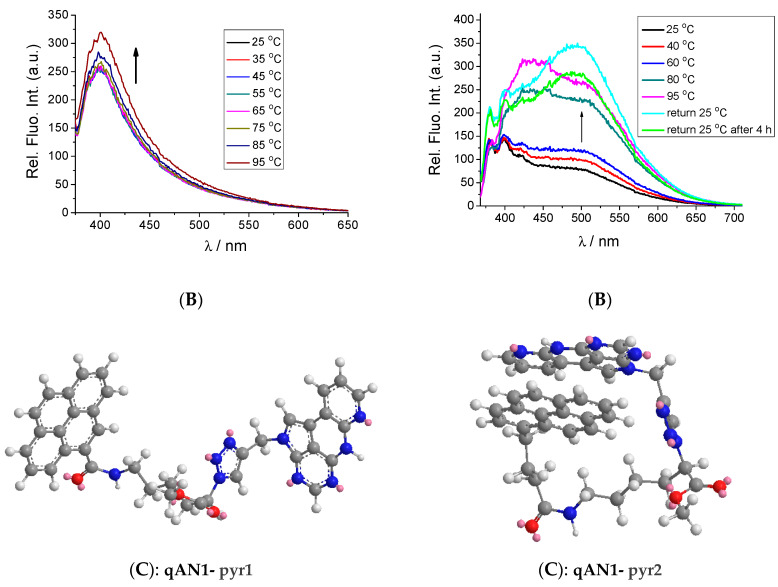
Comparison of **qAN1-pyr1** and **qAN1-pyr2** fluorescence spectra (λ_exc_ = 350 nm, *c* = 8 µM, pH 7.0, *I* = 0.05 M): (**A**) Concentration dependence of emission; (**B**) temperature dependence of emission. (**C**) Schematic presentation of intramolecular organization of **qAN1-pyr1** and **qAN1-pyr2** according to UV/Vis and fluorimetric data (supported by preliminary energy-minimization done in vacuum, Suppl. Info., Figure S19).

However, the fluorescence of the pyrene conjugates **qAN1-pyr1** and **qAN1-pyr2** differed significantly from **qAN1-AA** (Figure 2). Both compounds emitted with significantly lower intensity compared to **qAN1-AA**. From previous studies it is known that the quantum yield for the qAN1-monomer chromophore in aqueous solution is 18% [14] indicating quantum yields of **qAN1-pyr1** and **qAN1-pyr2** of approximately 1% and 3%, respectively. The emission maximum of **qAN1-pyr1** at λ_max_ = 400 nm suggests that the pyrene emission is dominant over **qAN1** emission (λ_max_ = 430 nm), which in this heterodimer appears to be almost completely quenched. Heating the solutions of both **qAN1-AA** (Suppl. Info. Figure S4) and **qAN1-pyr1** (Figure 3B left) induced only negligible emission change in accordance with the similarly negligible absorption changes observed (Suppl. Info. Figures S1 and S2).

Intriguingly, the fluorescence spectrum of **qAN1-pyr2** strongly differed from both **qAN1-AA** and **qAN1-pyr1** (Figure 2). The emission of **qAN1-pyr2** is characterized by two maxima: One at λ_max_ = 400–430 nm corresponding to the **qAN1-pyr1** emission and another, significantly red-shifted maximum (λ_max_ ≈ 500 nm) of similar intensity. The qAN1 fluorophore showed similar red-shifted emission (λ_max_ ≈ 500 nm) when incorporated in some ds-DNA oligonucleotides (Wranne et al. [16] and Figure 2) and that effect was attributed to qAN1 tautomerization in the excited state, caused by aromatic stacking interactions of qAN1 with adjacent base pairs. If the effect can be attributed to the same mechanism here that would suggest intramolecular stacking interactions between pyrene and qAN1 within one **qAN1-pyr2** molecule, resulting in exciplex-type emission. Another possible reason for the appearance of the λ_max_ at approximately 500 nm is pyrene-excimer emission [18,19,21], which is caused by two or more aromatically stacked pyrenes, thus presuming intermolecular interactions of two or more **qAN1-pyr2** molecules.

To further test both hypotheses, we studied the concentration dependent emission and noticed that the **qAN1-AA** and **qAN1-pyr1** emission intensities were proportional to concentration in a wide range of concentrations (up to 10 µM; Figure 3A left and Suppl. Info. Figure S4), whereas the **qAN1-pyr2** emission was proportional to concentration only up to 2 µM. At higher concentrations (*c* = 2–8 µM, Figure 3A right) the emission of **qAN1-pyr2** shows distinct non-linearity, implying intermolecular interactions.

Moreover, the heating of the **qAN1-pyr2** solution at high concentration (8 µM) resulted in a strong increase in the emission at λ_max_ = 495 nm (Figure 3B right). Returning to room temperature only partially restored the spectrum, even after four hours. This agrees well with the corresponding heating UV/Vis experiment (Suppl. Info. Figure S3); both UV/Vis and fluorescence results suggesting molecule aggregation/colloidization. The heating-induced aggregation is a well-known phenomenon for non-polar molecules and particularly hydrocarbons in aqueous solutions [43]. Thus, our results suggest that **qAN1-pyr2** at concentrations < 2 µM is mostly present in the intramolecularly stacked form, whereas at higher concentrations (>2 µM) or upon heating intermolecular dimers or larger aggregates are formed by stacking of pyrene chromophores, resulting in additional pyrene excimer emission (Figure 3B right).

The extensive difference in emissive response of two close analogues, **qAN1-pyr1** and **qAN1-pyr2**, could be correlated to and explained by the difference in linker length and rigidity, as schematically presented intramolecular aromatic stacking of a pyrene and **qAN1** for the longer and more flexible linker of **qAN1-pyr2** (Figure 3C right), in contrast to the stretched conformation of the shorter and more rigid linker of **qAN1-pyr1** (Figure 3C left).

### 2.3. Interactions with ds-DNA, ds-RNA and ss-RNA

With the insight from the investigations of the pure compounds regarding aggregation issues (vide supra), we decided to use compound concentrations < 2 µM when studying their interaction with various nucleic acid structures.

To study the interactions with DNA/RNA, several common types of DNA and RNA were chosen (Suppl. Info. Chart S1). Naturally occurring *calf thymus* (ct)-DNA represents a typical *B*-helix structure with a balanced ratio of GC-(48%) and AT-(52%) base pairs. Synthetic alternating polynucleotides poly (dGdC)_2_ and poly (dAdT)_2_ represent two extreme situations (only AT- or GC-basepairs, respectively), differing significantly in their secondary structures as well as in the availability of the minor groove for small molecule binding (the guanine amino group sterically hinders deep molecule penetration). For comparison between double stranded (ds) DNA and ds-RNA, poly rA- poly rU RNA was chosen as an *A*-helical structure characterized by a major groove available for the binding of bulky small molecules.

Furthermore, to study the ability of adenine–analogue **qAN1** to recognize complementary nucleobases, we also studied the single stranded synthetic ss-RNA polynucleotides poly G, poly A, poly U and poly C, each of them characterized by different properties. Adenine ss-RNA mimics 50-250 adenine nucleotides at the 3′ end of mRNA, poly G is related to guanine-rich sequences in both DNA and RNA, whereas poly C and poly U are significantly more flexible than purine-RNAs, and with less organized secondary structures.

#### 2.3.1. Fluorimetric Titrations

Due to the sensitivity of the method, we started studying the interaction between our compounds and nucleic acids by fluorescence measurements. Addition of any ds-RNA/RNA or ss-RNA in the range *r*_[**qAN1-AA**]/[polynucleotide base]_ = 1–0.01 induced small changes in the fluorescence spectrum of **qAN1-AA**, varying between 2–10% from the starting emission intensity (Suppl. Info. Figure S5). None of the titration experiments revealed any biologically relevant affinity even at 100-fold excess of any of polynucleotides, thus showing that **qAN1-AA** alone does not interact significantly with DNA or RNA.

**Figure 4:**
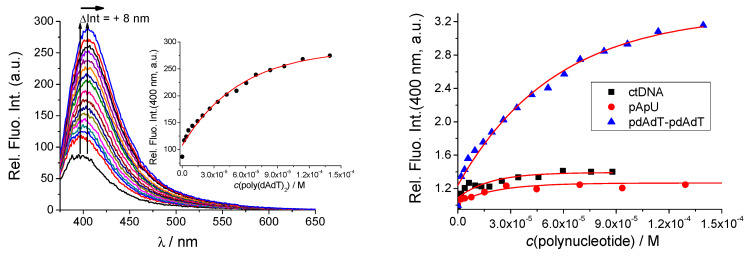
(**Left**): Fluorimetric titration of **qAN1-pyr1** (*c* = 1 × 10^−6^ M; λ_exc_ = 350 nm) with poly (dAdT)_2_; (**Right**): titrations with ds-DNA/RNA at 400 nm. Performed in sodium cacodylate buffer (pH 7, *I* = 0.05 M) at room temperature.

**Figure 5:**
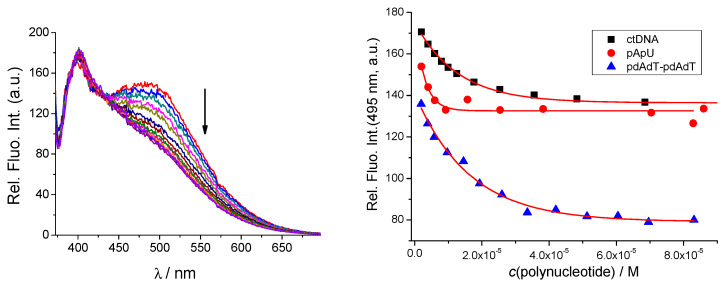
(**Left**): Fluorimetric titration of **qAN1-pyr2** (*c* = 1 × 10^−6^ M; λ_exc_ = 350 nm) with poly (dAdT)_2_; (**Right**): Titrations with ds-DNA/RNA at λ_em_ = 495 nm. Performed in sodium cacodylate buffer (pH 7, *I* = 0.05 M) at room temperature.

Fluorimetric titrations of the conjugates of **qAN1** and pyrene (**qAN1-pyr1** and **qAN1-pyr2**) with DNA/RNA revealed significant binding (Figure 4 and Figure 5, and Suppl. Info.). Moreover, the fluorimetric response was directly correlated to the length and rigidity of a linker between **qAN1** and pyrene (*cf*. Figure 4 and Figure 5). Fluorimetric titrations were processed by means of the Scatchard eq. (McGhee, vonHippel formalism) [44,45], to afford the binding constants and the density of binding sites (Table 1, log *K*_s_ and ratio *n*), as well as the relative emissive properties of complex formed (I/I_0_).

Detailed analysis of the fluorimetric results obtained for **qAN1-pyr1** and **qAN1-pyr2** binding to nucleic acids reveal that both analogues showed similar, moderate affinity toward ds-DNA and ds-RNA (except for **qAN1-pyr2**/ds-RNA, for which negligible emission change did not allow log *K_s_* determination). For both compounds the strongest emission change was observed for AT-dsDNA (Figure 4 and Figure 5 and Table 1), suggesting that DNA minor groove is preferential binding site. Namely, GC-DNA minor groove is sterically hampered and ds-RNA-major groove is much deeper and allows presence of much more water molecules (Suppl. Info. Chart S1), both characteristics exposing molecules to water and diminishing fluorescence change effect.

However, due to the basic difference in the intrinsic emission spectrum (only **qAN1-pyr2** exhibiting stacked pyrene-qAN1 emission at λ_max_ = 500 nm) opposite sign in emission changes was observed. The intramolecularly stacked **qAN1-pyr2** only revealed quenching of the stacked pyrene-qAN1 emission (λ_max_ = 500 nm) whereas the emission maximum at λ_max_ = 400–430 nm remained unchanged (Figure 5; Suppl. Info. Figures S9 and S10). In contrast, the non-stacked **qAN1-pyr1** emission (λ = 400–430 nm) increased significantly and underwent a slight red-shift upon addition of nucleic acids (Figure 4; Suppl. Info. Figures S6 and S7).

To shed more light on the thermodynamic properties and binding mode of the interaction between the compounds and the ds-DNA/RNA we performed thermal denaturation experiments. It is well established that upon heating duplexes of polynucleotides they dissociate into two single-stranded polynucleotides at a well-defined temperature (melting temperature *Tm*). Non-covalent binding of small molecules to duplex nucleic acids usually increases the thermal stability of duplexes, leading to an increase in *Tm* values [46]. For instance, if a pyrene analogue would intercalate into ds-DNA, a melting temperature increase of 5 °C or more would be expected, whereas a pyrene moiety interacting within a groove of a nucleic acid structure should result in a negligible stabilization effect [47].

Our studied compounds revealed only negligible stabilization of DNA or RNA duplexes (∆*Tm* = 0–1 °C, Suppl. Info. Table S2 and Figures S13–S15), which suggests that the compounds do not interact with the nucleic acids through intercalation, but rather by binding in the DNA/RNA grooves (in agreement with fluorimetric titration results above), through a combination of hydrophobic, van der Waals and other non-specific interactions, resulting in a moderate binding affinity.

**Figure 6:**
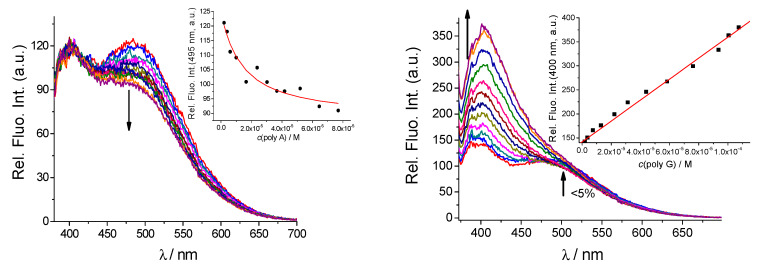
Fluorimetric titration of **qAN1-pyr2** (*c* = 1 × 10^−6^ M; λ_exc_ = 350 nm) with: (**Left**): poly A, Inset: emission change at 495 nm; (**Right**): poly G, Inset: emission change at 408 nm. Performed in sodium cacodylate buffer (pH 7, *I* = 0.05 M).

Further, addition of single stranded (ss)-RNAs induced different emission changes for the studied compounds (Figure 6, Suppl. Info. Figures S8–S12). Also, the binding constants (Table 1) were about an order of magnitude lower than obtained for most ds-polynucleotides.

The emission of **qAN1-pyr1** was only slightly changed by addition of any of ss-RNA (<15%) with no apparent selectivity between different RNA sequences (Supp. Info. Figure S8).

However, the fluorimetric response of **qAN1-pyr2** is stronger and different in comparison to **qAN1-pyr1** and the change in spectral shape is strongly dependent on the nature of the nucleobase for ss-RNA (Figure 6).

Firstly, the addition of poly U (Watson–Crick complementary base to **qAN1**) resulted in emission quenching of the **qAN1-pyr2** at λ_max_ = 500 nm (Figure S12, 25% quenching), significantly stronger in comparison to the W-C non-complementary poly C (Figure S11, 5% quenching). Strong quenching was observed also upon addition of poly A (Figure 6 left, 25% quenching). Intriguingly, the change in **qAN1-pyr2** emission at λ = 400–430 nm (Figure S12 and Figure 6 left) was minor, suggesting that the binding process between **qAN1-pyr2** and poly U or poly A mainly involves an intramolecularly stacked pyrene-qAN1 fluorophore (λ_max_ = 500 nm). Since qAN1 is an adenine analogue, it seems feasible that Uracil-qAN1 (Watson–Crick) or Adenine-qAN1 (reverse Hoogsteen [48]) pair is formed, additionally stabilized by aromatic stacking by adjacent pyrene moieties. Such a duplex-like structure would show slight resemblance with the previously studied duplex structure containing qAN1 (Wranne et al. [16], Figure 2: TA-sequence), which also showed the emission at λ_max_ = 500 nm. Accordingly, inability of poly C to form H-bonds with qAN1 and small aromatic surface of cytosine did not support binding and thus resulted in negligible fluorimetric change.

The most intriguingly, a strong emission increase of **qAN1-pyr2** at λ = 400–430 nm is observed exclusively upon poly G addition, accompanied by negligible change at λ_max_ = 500 nm (Figure 6 right). This suggested that structure of **qAN1-pyr2**/poly G complex is significantly different compared to the complex with poly A or poly U, likely because guanine bases of the poly G cannot form efficient H-bond pairing with qAN1 due to the unfavorable order of H-bond donors and H-bond acceptors. Taking into account that emission of reference compound **qAN1-AA** did not change upon any polynucleotide addition up to 100 µM range, strong increase of emission at λ_max_ = 408 nm suggests at least partial release of either pyrene or qAN2 from self-folded (and self-quenched) **qAN1-pyr2** form. Negligible change of emission at 500 nm could be result of pyrene stacking with guanines compensating the release of free qAN2.

However, such an unstacking of **qAN1-pyr2** would have a thermodynamic price, and also would not include H-bonding which could be present for both poly U and poly A. This would result in the significantly lower affinity of **qAN1-pyr2** that we find towards poly G (log *K_s_* < 3) in comparison to complexes with poly U and poly A (log *K_s_* ≈ 5).

#### 2.3.2. Circular Dichroism (CD) Experiments

In order to get insight into the changes of polynucleotide properties induced by the small molecule binding, we chose CD spectroscopy as a sensitive method for the study of secondary structure conformational changes [49]. In addition, achiral small molecules can acquire an induced CD spectrum (ICD) upon binding to polynucleotides, which could give useful information about modes of interaction [49,50].

In our investigations, however, the addition of our compounds did not induce any significant changes in CD spectrum of any ds-DNA/RNA. Neither was any measurable ICD effect > 300 nm observed (Suppl. Info. Figures S16–S18). These results along with the negligible thermal denaturation effect (Table S2) point in the direction that DNA/RNA double helices are not disturbed upon small molecule interaction, thus speaking against an intercalative binding mode and suggesting the grooves of ds-polynucleotides as binding sites. However, absence of any measurable ICD effect in the 300–350 nm region (at which both qAN1 and pyrene absorb) argues against typical, uniformly-oriented groove binding. Negligible ICD effect for the groove-binding compounds would imply either: (a) Chromophores are not homogeneously oriented within binding site with respect to a chiral axis of the polynucleotide, or (b) transition vectors of qAN1 and pyrene responsible for the absorbance at 300–350 nm yield opposite ICD signs upon binding to DNA/RNA, resulting in ICD bands of negligible intensity.

## 3. Conclusions

The here designed and prepared novel amino acid-fluorescent nucleobase derivative, **qAN1-AA**, allows several conjugation strategies within peptide-backboned systems (PNAs or similar), either by direct incorporation of **qAN1-AA** by peptide bond formation or via a “click”-related route: First introducing azide-amino acid at targeted peptide position and then “clicking” the nucleobase at any convenient reaction point. In addition, our novel propargyl-**qAN1** precursor can also be “clicked” to any other azide-based building block.

We find that the amino acid-fluorescent nucleobase derivative (**qAN1-AA**) does not interact with DNA/RNA at biologically relevant conditions. However, linking the **qAN1-AA** with pyrene yielded conjugates (**qAN1-pyr1, qAN1-pyr2)** capable of interacting with various DNA/RNA (1-10 µM affinity), with a fluorescent response that senses differences in secondary structure and nucleobase composition of nucleic acid targets.

The difference in linker length between the pyrene conjugates strongly influenced their spectroscopic properties in aqueous solution. The longer and more flexible linker directed **qAN1-pyr2** into a intramolecularly stacked qAN1/pyrene conformation, yielding strongly red-shifted emission (λ = 500 nm) in comparison to both free pyrene or qAN1, whereas the shorter and more rigid linker of **qAN1-pyr1** resulted in a non-stacked conformation characterized by the emission of free pyrene at λ = 400–430 nm. This intrinsic conformational difference was the basis of different fluorimetric responses of the studied conjugates upon binding to various DNA or RNA. We find evidence that the conjugates bind to ds-DNA/RNA grooves with essentially no impact on polynucleotide double helix stability or chirality.

The most pronounced change in emission of both **qAN1-pyr1** and **qAN1-pyr2**, was observed for AT-DNA addition. That is in accordance with the AT-DNA minor groove availability to small molecule binding [51]. It has previously been established that the AT-DNA minor groove has ideal dimensions for insertion of small molecules [52] (Suppl. Info. Chart S1). This seems also to be the case not only to flexible **qAN1-pyr1** but also for the self-folded dual aromatic system as **qAN1-pyr2**. On the contrary, the GC-DNA minor groove is sterically hindered by protruding amino groups of guanines, leaving small molecule more exposed to water and thus its emission less changed [51,52]. The ds-RNA minor groove is too shallow and broad for efficient small molecule binding and RNA major groove can accommodate these small molecules but due to large depth (Suppl. Info. Chart S1). Cantor, C.R. et al. and Egli, M. et al., [52,53] does not exclude water molecules as efficiently as AT-DNA minor groove, resulting in less efficient fluorescence change.

Most importantly, the intramolecularly pre-oriented **qAN1-pyr2** clearly differentiated between ss-RNAs by opposite emission response. This is most likely based on H-bonding complementarity: Watson–Crick complementary poly U or reverse Hoogsteen-complementary [48] poly A to the qAN1 moiety yielded efficient emission quenching at 500 nm, whereas non-complementary poly C did not influence emission and the non-complementary poly G yielded an strong emission increase at 408 nm (likely to bis-intercalation of **qAN1-pyr2**, see discussion under Figure 6). Unlike **qAN1-pyr2**, the non-intramolecularly folded **qAN1-pyr1** emission did not change significantly upon addition of any ss-RNA, clearly stressing the importance of **qAN1-pyr2** intramolecular pre-organization for variation in emission response upon interaction.

Chromophores and especially fluorophores which can exist in multiple conformational states with the possibility to change between them based on binding to various targets, have high potential for the use in molecular sensing applications. For that reason here presented **qAN1-pyr2** shows promise as a specific fluorimetric probe for non-covalent sensing of poly G and also as a good lead compound for further development of probes targeting uracil- or adenine- DNA abasic sites, i.e., DNA abasic sites as products of chemotherapy or environmental impacts are promising targets for aryl-nucleobase conjugates [54] and particularly pre-organized, self-folded analogues has previously shown high selectivity [55,56,57,58]. Therefore, we envision that **qAN1-pyr2** and its future derivatives could not only have sensor applications but also, simultaneously, display therapeutic properties.

## 4. Materials and Methods

### 4.1. General Information

Solvents were distilled from appropriate drying agents shortly before use. TLC was carried out on TLC Silica gel 60 F254 Plastic sheets and preparative thin layer (2 mm) chromatography was done on Merck 60 F254 plates (Merck KGaA, Darmstadt, Germany). NMR spectra were recorded on AV600 and AV300 MHz spectrometers (Bruker BioSpin GmbH, Rheinstetten, Germany), operated at 600.13 or 300.13 MHz for ^1^H nuclei and at 150.92 MHz or 75.46 MHz for ^13^C, using DMSO-*d*_6_ (δ_H_: 2.50 ppm, δ_C_: 39.52 ppm) or CDCl_3_ (δ_H_: 7.26 ppm, δ_C_: 77.16 ppm) as the internal standard. Mass spectrometry was performed on an Agilent 6410 Triple Quad mass spectrometer (Agilent Technologies, Santa Clara, CA, USA). High-resolution mass spectra (HRMS) were obtained using a Q-TOF2 hybrid quadrupole time-of-flight mass spectrometer (Micromass, Cary, NC, USA).

### 4.2. Synthesis

*N_3_-Lys(Boc)-OH* (**3**)*:* H-Lys(Boc)-OH (**2**, 1.065 g, 4.32 mmol) was dissolved in a biphasic mixture of H_2_O (21 mL), MeOH (42 mL), and CH_2_Cl_2_ (35 mL). CuSO_4_·5H_2_O (6.9 mg, 0.028 mmol) and azide **1** (2.72 g, 12.96 mmol) were added, and the mixture was adjusted to pH 9 with aq K_2_CO_3_ solution. After stirring vigorously for 15 h, the reaction mixture was diluted with CH_2_Cl_2_ (45 mL) and the aqueous phase was isolated. The organic phase was extracted with satd. NaHCO_3_ (2 × 50 mL). The combined aqueous extracts were washed with Et_2_O (2 × 50 mL), acidified to pH 2 with concentrated HCl, and extracted with Et_2_O (3 × 60 mL). The organic extracts were dried over Na_2_SO_4_ and concentrated in vacuo to give azide **3** (1.199 g, 98%) as a transparent oil: ^1^H NMR (300 MHz, DMSO-*d*_6_) *δ*/ppm: 13.26 (br. s, 1H, COOH), 6.78 (t, *J* = 5.2 Hz, 1H, NH), 3.57–3.23 (m, 3H, CH, CH_2_), 2.89 (q, *J* = 6.3 Hz, 2H, CH_2_), 1.82–1.49 (m, 2H, CH_2_), 1.37 (br. s, 11H, CH_2_, C(CH_3_)_3_); ^13^C NMR (75 MHz, DMSO-*d*_6_) *δ*/ppm: 173.0 (s, C=O), 155.7 (s, C=O Boc), 77.4 (s, C(CH_3_)_3_), 64.2 (d, CH), 39.9 (t, CH_2_NH), 31.6 (t, CH_2_), 29.3 (t, CH_2_), 28.3 (q, C(CH_3_)_3_), 23.5 (t, CH_2_). ESI-MS: *m/z* calcd. for C_11_H_21_N_4_O_4_ [M+H]^+^ 272.15, found 272.3.

*Propargylated qAN1* (**4**): To a suspension of **qAN1** (57 mg, 0.184 mmol) and K_2_CO_3_ (25.4 mg, 0.184 mmol) in DMF (3 mL) propargyl bromide (0.04 mL, 0.369 mmol) was added. The reaction mixture was stirred in the dark at room temperature for 4 days. The solvent was evaporated under reduced pressure and the resulting solid was treated with CH_2_Cl_2_ and filtered. The filtrate was concentrated under reduced pressure and the residue was purified by preparative chromatography (CH_2_Cl_2_/MeOH, 20:1) to give the product **4** (61 mg, 95%) as a light yellow solid: *R*_f_ = 0.52 (CH_2_Cl_2_/MeOH, 20:1); ^1^H NMR (300 MHz, DMSO-*d*_6_) *δ*/ppm: 8.31 (s, 1H, H-4), 8.21–8.13 (m, 2H, H-8, H-10), 7.63 (s, 1H, H-1), 7.21 (dd, *J* = 7.7, 4.9 Hz, 1H, H-9), 5.08 (d, *J* = 2.5 Hz, 2H, CH_2_), 3.52 (t, *J* = 2.5 Hz, 1H, HC≡), 1.60 (s, 9H, C(CH_3_)_2_); ^13^C NMR (75 MHz, DMSO-*d*_6_) *δ*/ppm: 155.4 (d, C4), 152.0 (s, C=O), 149.5 (s, C15 or C11), 149.3 (s, C11 or C15), 146.9 (s, C16), 146.1 (d, C8), 132.8 (d, C10), 120.3 (d, C-9), 116.4 (s, C12), 116.2 (d, C1), 107.8 (s, C13 or C14), 106.4 (s, C14 or C13), 85.2 C(CH_3_)_3,_ 78.5 (s, C≡CH), 76.1 (s, HC≡C-), 33.8 (t, CH_2_)_,_ 27.3 (q, C(CH_3_)_3_). ESI-MS: *m/z* calcd. for C_19_H_18_N_5_O_2_ [M+H]^+^ 348.38, found 348.9.

*qAN1 protected conjugate* (**5**): To a solution of **4** (24 mg, 0.07 mmol) in anhydrous CH_2_Cl_2_ (1.5 mL), azide **3** (19 mg, 0.07 mmol), CuI (27 mg, 0.14 mmol) and DIPEA (19 µL, 0.21 mmol) were added and stirred overnight. After evaporation of the solvent, CH_2_Cl_2_ and MeOH were added and the resulting solid was filtered off. The filtrate was evaporated and the residue was purified by preparative chromatography (CH_2_Cl_2_/MeOH, 9:1) to give 32 mg (74%) of yellow **5**: *R*_f_ = 0.33 (CH_2_Cl_2_/MeOH, 9:1); ^1^H NMR (600 MHz, DMSO-*d*_6_) *δ*/ppm: 8.30 (s, 1H, H-4), 8.16–8.15 (m, 1H, H-8), 8.12–8.11 (m, 1H, H-10), 8.05 (s, 1H, =CH), 7.58 (s, 1H, H-1), 7.18–7.16 (m, 1H, H-9), 6.69 (m, 1H, NH), 5.47–5.41 (m, 2H, CH_2_), 4.69 (s, 1H, CH), 2.81–2.80 (m, 2H, CH_2_), 2.02–1.82 (m, 2H, CH_2_), 1.59 (m, 9H, C(CH_3_)_3_), 1.35–1.08 (m, 13 H, 2x CH_2,_ C(CH_3_)_2_); ^13^C NMR (151 MHz, DMSO-*d*_6_) *δ*/ppm: 155.5, 155.2, 152.0, 149.5, 149.4, 147.0, 145.9, 141.5, 132.6, 122.5, 120.2, 116.8, 116.4, 114.6, 107.4, 106.4, 85.0, 77.2, 33.2, 31.2, 28.9, 28.2, 27.3, 23.4, 22.0, 13.9. ESI-MS: *m/z* calcd. for C_30_H_37_N_9_O_6_ [M+H]^+^ 620.67, found 620.9.

*qAN1-AA:* Compound **5** (12 mg) was dissolved in a 1:1 mixture of TFA/CH_2_Cl_2_ (4 mL) and stirred at room temperature overnight. After removal of the remaining TFA under reduced pressure, **qAN1-AA** (13 mg, 100%) was isolated as a yellow powder. ^1^H NMR (300 MHz, DMSO-*d*_6_) *δ*/ppm: 8.15–7.98 (m, 3H, H-4, H-8, H-10), 7.64 (s, 2H, NH_2_), 7.49–7.39 (m, 1H, H-1), 7.30 (s, 1H, =CH), 7.13–6.96 (m, 2H, H-9, NH), 5.50 (br. s., 2H, CH_2_), 4.13 (s, 1H, CH), 2.73 (m, 2H, CH_2_), 2.28–2.22 (m, 2H, CH_2_), 1.51 (s, 2H, CH_2_), 0.87–0.84 (m, 2 H, CH_2_); ^13^C NMR (75 MHz, DMSO-*d*_6_) *δ*/ppm: 158.2, 157.8, 155.7, 154.6, 151.6, 146.6, 146.3, 133.5, 131.9, 118.6, 116.2, 114.5, 109.4, 55.9, 52.8, 38.4, 30.4, 26.2, 22.3. HRMS (MALDI-TOF/TOF): *m/z* calcd. for C_20_H_22_N_9_O_2_ [M+H]^+^ 420.1896, found 420.1893.

*N_3_-Lys-OH·HCl* (**6**): To a solution of azide **3** (510 mg, 1.87 mmol) 3M methanolic HCl (2.5 mL) was added and stirred for 4 h at room temperature. The solvent was evaporated and compound **6** (414 mg) was isolated in quantitative yield. ^1^H NMR (300 MHz, CDCl_3_) *δ*/ppm: 8.06 (s, 3H, NH_3_), 3.95–3.91 (m, 1H, CH), 3.78 (3, 3H, OCH_3_), 3.02 (s, 2H, CH_2_), 1.92–1.43 (m, 6H, 3xCH_2_). ESI-MS: *m/z* calcd. for C_7_H_13_N_3_O_2_[M+H]^+^ 171.20, found 171.3.

*Azido-pyrene ligand* (**7**): Azide **6** (55 mg, 0.25 mmol) and 1-pyrenecarboxylic acid (61 mg, 0.25 mmol) were dissolved in dry DMF (2.5 mL) under argon and HOBt (33.5 mg, 0.25 mmol), HCTU (102.5 mg, 0.25 mmol) and dry Et_3_N (138 µL, 1.0 mmol) were added. The reaction was stirred at room temperature overnight. Product **7** (91 mg, 87%) was isolated by preparative chromatography (CH_2_Cl_2_/CH_3_OH 20:1) as a light yellow oil. *R*_f_ = 0.85 (CH_2_Cl_2_/CH_3_OH 20:1); ^1^H NMR (600 MHz, DMSO-*d*_6_) *δ*/ppm: 8.70 (t, *J* = 5.3 Hz, 1H), 8.48 (d, *J* = 9.2 Hz, 1H), 8.36–8.32 (m, 3H, Py), 8.26–8.22 (m, 3H, Py), 8.13 – 8.10 (m, 2H, Py), 4.34 (dd, *J* = 8.2, 5.0 Hz, 1H, CH), 3.74 (s, 3H, OCH_3_), 3.42 (dd, *J* = 12.7, 6.7 Hz, 2H, CH_2_), 1.90–1.73 (m, 2H, CH_2_), 1.70–1.63 (m, 2H, CH_2_), 1.53–1.48 (m, 2H, CH_2_); ^13^C NMR (75 MHz, DMSO-*d*_6_): 170.8, 168.8, 132.3, 131.5, 130.7, 130.2, 128.2, 128.0, 127.7, 127.2, 126.6, 125.8, 125.6, 125.1, 124.7, 124.4, 123.8, 123.7, 61.2, 52.5, 38.9, 30.5, 28.6, 22.7. ESI-MS: *m/z* calcd. for C_24_H_23_N_4_O_3_ [M+H]^+^ 415.46, found 415.2.

*qAN1-pyr1:* To a solution of alkyne **4** (12 mg, 0.03 mmol) in anhydrous CH_2_Cl_2_ (2 mL), azido-pyrene **7** (14 mg, 0.03 mmol), CuI (13 mg, 0.07 mmol) and DIPEA (9 µL, 0.1 mmol) were added and stirred overnight. After evaporation of the solvent, CH_2_Cl_2_ was added and the resulting solid was filtered off. The filtrate was purified by preparative chromatography (CH_2_Cl_2_/CH_3_OH 20:1) which yielded 15 mg (76%) of Boc-deprotected final product **qAN1-pyr1** as a yellow powder. *R*_f_ = 0.45 (CH_2_Cl_2_/MeOH, 20:1); ^1^H NMR (300 MHz, DMSO-*d*_6_) *δ*/ppm: 11.07 (br s, 1H, NH), 8.64 (t, *J* = 5.4 Hz, 1H), 8.43–8.02 (m, 12H, H-4, H-8, H-10, NH, Py, =CH), 7.87 (d, *J* = 7.1 Hz, 1H, Py), 7.33 (s, 1H, H-1), 7.00–6.96 (m, 1H, H-9), 5.66–5.36 (m, 3H, CH + CH_2_), 3.68 (s, 3H, OCH_3_), 2.73–2.50 (m, 2H, DMSO + CH_2_), 2.33–2.26 (m, 2H, CH_2_), 1.65–1.23 (m, 4H, 2xCH_2_); ^13^C NMR (75 MHz, DMSO-*d*_6_) *δ*/ppm: 177.5, 169.7, 169.6, 169.3, 169.2, 155.5, 146.7, 138.0, 132.7, 132.5, 131.9, 131.2, 130.6, 128.7, 128.5, 128.1, 127.6, 127.0, 126.2, 126.0, 125.6, 125.5, 125.1, 124.8, 124.2, 124.1, 120.2, 62.2, 53.3, 35.9, 31.2, 31.1, 28.8, 23.3. HRMS (MALDI-TOF/TOF): *m/z* calcd. for C_38_H_32_N_9_O_3_[M+H]^+^ 662.2628, found 662.2639.

*Azido-pyrene ligand* (**8**): Azide **6** (58 mg, 0.26 mmol) and 1-pyrenebutyric acid (75 mg, 0.26 mmol) were dissolved in dry DMF (2 mL) under argon and HOBt (35 mg, 0.26 mmol), HCTU (108 mg, 0.26 mmol) and dry Et_3_N (145 µL, 1.04 mmol) were added. The reaction was stirred at room temperature overnight. Pyrene conjugate **8** (110 mg, 92%) was isolated by preparative chromatography (CH_2_Cl_2_/CH_3_OH 20:1) as a yellow oil. *R*_f_ = 0.85 (CH_2_Cl_2_/CH_3_OH 20:1); ^1^H NMR (600 MHz, DMSO-*d*_6_) *δ*/ppm: 8.38 (d, *J* = 9.3 Hz, 1H, NH), 8.27 (t, *J* = 7.7 Hz, 2H, Py), 8.22 (dd, *J* = 8.5, 4.7 Hz, 2H, Py), 8.15–8.10 (m, 2H, Py), 8.05 (t, *J* = 7.6 Hz, 1H, Py), 7.94 (d, *J* = 7.8 Hz, 1H, Py), 7.83 (t, *J* = 5.6 Hz, 1H, Py), 4.23 (dd, *J* = 8.3, 5.0 Hz, 1H, CH), 3.68 (s, 3H, OCH_3_), 3.31–3.30 (m, 2H, CH_2_), 3.06 (dd, *J* = 12.7, 6.7 Hz, 2H, CH_2_), 2.22 (t, *J* = 7.3 Hz, 2H, CH_2_), 2.04–1.99 (m, 2H, CH_2_), 1.77–1.62 (m, 2H, CH_2_), 1.43–1.39 (m, 2H, CH_2_), 1.35–1.31 (m, 2H, CH_2_). ^13^C NMR (151 MHz, DMSO-*d*_6_) *δ*/ppm: 171.7, 170.7, 136.5, 130.9, 130.4, 129.3, 128.1, 127.5, 127.4, 127.2, 126.5, 126.1, 124.9, 124.7, 124.2, 124.1, 123.5, 61.1, 52.4, 38.1, 35.0, 32.2, 30.4, 28.6, 27.6, 22.5. ESI-MS: *m/z* calcd. for C_27_H_29_N_4_O_3_ [M+H]^+^ 457.54, found 457.2.

*Protected qAN1-amino acid-pyrene conjugate* (**9**): To a solution of alkyne **4** (18 mg, 0.05 mmol) in anhydrous CH_2_Cl_2_ (2 mL), azido-pyrene **8** (24 mg, 0.05 mmol), CuI (19 mg, 0.10 mmol) and DIPEA (14 µL, 0.15 mmol) were added and stirred overnight. After evaporation of the solvent, CH_2_Cl_2_ was added and the resulting solid was filtered off. The filtrate was evaporated and MeOH was added which yielded the Boc-protected conjugate **9** (38 mg, 92%) as a yellow powder: *R*_f_ = 0.55 (CH_2_Cl_2_/MeOH, 20:1); ^1^H NMR (600 MHz, DMSO-*d*_6_) *δ*/ppm: 8.35–7.99 (m, 12H, H-4, H-8, H-10, Py, =CH), 7.90 (d, *J* = 7.7 Hz, 1H, Py), 7.77–7.73 (m, 1H, NH), 7.50 (s, 1H, H-1), 7.19–7.15 (m, 1H, H-9), 5.59–5.44 (m, 3H, CH + CH_2_), 3.63 (s, 3H, OCH_3_), 3.02–2.96 (m, 2H, CH_2_), 2.73–2.50 (m, 2H, DMSO + CH_2_), 2.28–2.13 (m, 4H, 2xCH_2_), 1.98–1.94 (m, 2H, CH_2_), 1.58 (m, 9H, C(CH_3_)_3_), 1.44–0.96 (m, 4H, 2x CH_2_); ^13^C NMR (75 MHz, DMSO-*d*_6_) *δ*/ppm: 171.7, 169.1, 162.6, 151.8, 149.4, 149.3, 146.4, 146.0, 136.5, 132.7, 130.8, 130.3, 129.4, 128.1, 127.5, 127.4, 127.2, 126.4, 126.0, 124.87, 124.85, 124.8, 124.7, 124.2, 124.1, 123.4, 122.0, 120.2, 116.8, 116.3, 107.5, 103.6, 85.1_,_ 61.9, 52.7, 37.9, 34.9, 32.2, 30.4, 28.3, 27.5, 27.3, 22.5. ESI-MS: *m/z* calcd. for C_46_H_46_N_9_O_5_ [M+H]^+^ 804.91, found 804.3.

*qAN1-pyr2*: Conjugate **9** (15 mg) was dissolved in CH_2_Cl_2_ (4 mL) and applied to the preparative plates in solvent system CH_2_Cl_2_/CH_3_OH 20:1, which yielded 10 mg (82%) of final product **qAN1-pyr2** as a yellow powder. *R*_f_ = 0.5 (CH_2_Cl_2_/MeOH, 20:1); ^1^H NMR (300 MHz, DMSO-*d*_6_) *δ*/ppm: 8.35–8.00 (m, 13H, H-4, H-8, H-10, NH, Py, =CH), 7.90 (d, *J* = 7.8 Hz, 1H, Py), 7.78 (t, 1H, NH), 7.38 (s, 1H, H-1), 7.10–7.03 (m, 1H, H-9), 5.50 (t, *J* = 7.5 Hz, 1H, CH), 5.38 (s, CH_2_), 3.63 (s, 3H, OCH_3_), 3.53–3.24 (m, 2H, H_2_O + CH_2_), 3.00–2.98 (m, 2H, CH_2_), 2.19–2.12 (m, 4H, 2xCH_2_), 2.02–1.94 (m, 2H, CH_2_), 1.42–1.34 (m, 2H, CH_2_), 1.05–0.82 (m, 2H, CH_2_); ^13^C NMR (75 MHz, DMSO-*d*_6_) *δ*/ppm: 171.8, 169.2, 155.6, 153.8, 151.7, 145.2, 145.2, 136.5, 132.9, 130.9, 130.4, 129.3, 128.1, 127.5, 127.4, 127.2, 126.5, 126.1, 124.9, 124.8, 124.2, 124.1, 123.7, 123.5, 118.7, 116.9, 114.9, 109.9, 61.9, 52.8, 37.9, 35.0, 32.3, 30.5, 29.0, 28.4, 27.6, 22.6. HRMS (MALDI-TOF/TOF): *m/z* calcd. for C_41_H_37_N_9_O_3_Na[M+Na]^+^ 726.2917, found 726.2954.

### 4.3. Study of DNA/RNA Interactions

All measurements were performed in aqueous buffer solution (pH = 7.0, *I* = 0.05 M, sodium cacodylate/HCl buffer). The UV-Vis spectra were recorded on a Varian Cary 100 Bio spectrometer and fluorescence spectra were recorded on a Varian Cary Eclipse fluorimeter in quartz cuvettes (path length: 1 cm).

Polynucleotides were purchased as noted: poly dAdT–poly dAdT, poly A–poly U, poly A, poly G, poly C, poly U (Sigma), *calf thymus* (ct)-DNA (Aldrich) and dissolved in sodium cacodylate buffer, *I* = 0.05 M, pH = 7.0. The ct-DNA was additionally sonicated and filtered through a 0.45 mm filter to obtain mostly short (ca. 100 base pairs) rod-like B-helical DNA fragments [59]. Polynucleotide concentration was determined spectroscopically [60] as the concentration of phosphates (corresponds to *c*(nucleobase)).

In fluorimetric experiments an excitation wavelength of λ_exc_ = 342 nm was used to avoid absorption of excitation light by added polynucleotides. Fluorimetric titrations were performed by adding portions of polynucleotide solution into the solution of the studied compound being studied (*c* = 1 × 10^–6^ M). After mixing polynucleotides with the compound, equilibrium was reached in less than 120 s, which was checked by repeatedly collecting emission spectra after each addition until equilibrium is reached. Fluorescence spectra were collected using an excess of DNA/RNA (r_[**dye**]/[DNA]_ < 0.3) to assure one dominant binding mode. To obtain binding constants (*Ks*), titration data were processed by means of non-linear fitting to the Scatchard equation (McGhee, von Hippel formalism) [44,45] which gave values of the ratio of [bound compound]/[polynucleotide] in the range 0.1–0.3, but for easier comparison, all *Ks* values were re-calculated for the fixed *n* = 0.2 (for ds-DNA/RNA) or 0.5 (for ss-RNA). Calculated values for Ks have satisfactory correlation coefficients (>0.99).

Circular dichroism (CD) spectra were recorded on JASCO J-815 spectropolarimeter at room temperature using 1 cm path quartz cuvettes with a scanning speed of 200 nm/min (an average of 3 accumulations). A buffer background was subtracted from each spectrum. CD experiments were performed by adding portions of compound stock solution into the solution of the polynucleotide (*c* = 2 × 10^–5^ M).

Thermal melting experiments were performed on a Varian Cary 100 Bio spectrometer in quartz cuvettes (1 cm). The measurements were done in aqueous buffer solution at pH 7.0 (sodium cacodylate buffer, *I* = 0.05 M). Thermal melting curves for ds-DNA, ds-RNA and their complexes with dyes were determined by monitoring the absorption change at 260 nm as a function of temperature [46]*. Tm* values are the midpoints of the transition curves determined from the maximum of the first derivative and checked graphically by the tangent method. The ∆*Tm* values were calculated subtracting *Tm* of the free nucleic acid from *Tm* of the complex. Every ∆*Tm* value here reported was the average of at least two measurements. The error in ∆*Tm* is ± 0.5 °C.

## Figures and Tables

**Table 1:** Binding constants (log *K*_s_) ^a^ and emission change (I/I_0_) ^b^ calculated from the fluorescence titrations of compounds with ds- and ss- polynucleotides at pH 7.0 (sodium cacodylate buffer, *I* = 0.05 M).

	qAN1-AA log*K_s_*/I/I_0_^b^	qAN1-pyr1 log*K_s_*/I/I_0_^b^	qAN1-pyr2 log*K_s_*/I/I_0_^b^
ct-DNA	>0.95 ^d^	5.7/1.3	5.9/0.7
poly (dAdT)_2_	>0.95 ^d^	5.0/3.3	5.7/0.5
poly A-poly U	1 ^d^	5.5/1.3	>0.95 ^d^
poly G	>0.95 ^d^	5 ^e^/1.1	<3/>2 ^c^
poly A	1 ^d^	5 ^e^/1.1	5.2/0.7
poly C	1 ^d^	5 ^e^/1.2	>0.95 ^d^
poly U	1 ^d^	5 ^e^/1.1	4.7/0.6

^a^ Processing of titration data by means of Scatchard equation [44,45] gave similar values of ratio *n*[bound compd]/[polynucleotide base]. For easier comparison, all log *K_s_* values were re-calculated for fixed *n* = 0.2 for all dsDNA/RNA and *n* = 0.5 for ss-RNA. Correlation coefficients were > 0.99 for all calculated log *K_s_* values. ^b^ *I*_0_—starting fluorescence intensity of dye; *I—*fluorescence intensity of dye/polynucleotide complex calculated by Scatchard equation at the corresponding emission maximum [44]. ^c^ Linear dependence of emission change to *c*_polynucleotide_ allowed only estimation of log*K_s_*. ^d^ Too small emission changes for processing by Scatchard eq. ^e^ Estimate of log *K_s_* due to small emission changes.

## Supplementary Materials

Supplementary Materials are available online at https://www.mdpi.com/1420-3049/25/9/2188/s1, Additional characterization data, DNA/RNA binding data.
