# Supplementary material for: Flexibility and Preorganization of Fluorescent Nucleobase-Pyrene Conjugates Control DNA and RNA Recognition
Source: Molecules. 2020 May 7;25(9):2188. doi: 10.3390/molecules25092188 (PMC7248712; doi:10.3390/molecules25092188)
Supplement: Supplementary file 1 [file molecules-25-02188-s001.pdf]

Supporting information to Ban et al:

Flexibility and preorganization of fluorescent nucleobase - pyrene conjugates control DNA and RNA recognition

Željka Ban[1], Josipa Matić[1], Biserka Žinić[1], Anders Foller Füchtbauer[2], L. Marcus Wilhelmsson[2], Ivo Piantanida[1]

[1] Laboratory for Biomolecular Interactions and Spectroscopy, Division of Organic Chemistry & Biochemistry, Ruđer Bošković Institute, HR 10002 Zagreb, Croatia.

[2] Department of Chemistry and Chemical Engineering/Chemistry and Biochemistry, Chalmers University of Technology, Gothenburg S-41296, Sweden

Contents

Properties of studied DNA and RNA S2

UV/Vis and fluorescence spectra S3-S7

Fluorimetric titrations S8-S13

Thermal denaturation experiments S13-S14

Circular dichroism.......................................S Error! Bookmark not defined.**-S16**

Structural aspects S17

Properties of studied DNA and RNA

Polynucleotides were purchased as noted: poly dGdC – poly dGdC, poly dAdT – poly dAdT, poly A – poly U, poly A, poly G, poly C, poly U (Sigma), *calf thymus* (ct)-DNA (Aldrich) and dissolved in sodium cacodylate buffer, I = 0.05 M, pH=7.0. The ct-DNA was additionally sonicated and filtered through a 0.45 mm filter to obtain mostly short (ca. 100 base pairs) rod-like B-helical DNA fragments.[1]

Chart S1. Groove widths and depths for selected nucleic acid conformations [2,3].

Structure type	Groove width [Å]		Groove depth [Å]	
	major	minor	major	minor
[a] poly rA – poly rU	3.8	10.9	13.5	2.8
[b] poly dGdC – poly dGdC	13.5	9.5	10.0	7.2
[b] poly dAdT – poly dAdT	11.2	6.3	8.5	7.5

[a] A - helical structure (e.g. A-DNA)

[b] B - helical structure (e.g. B-DNA)

UV/Vis and fluorescence spectra:

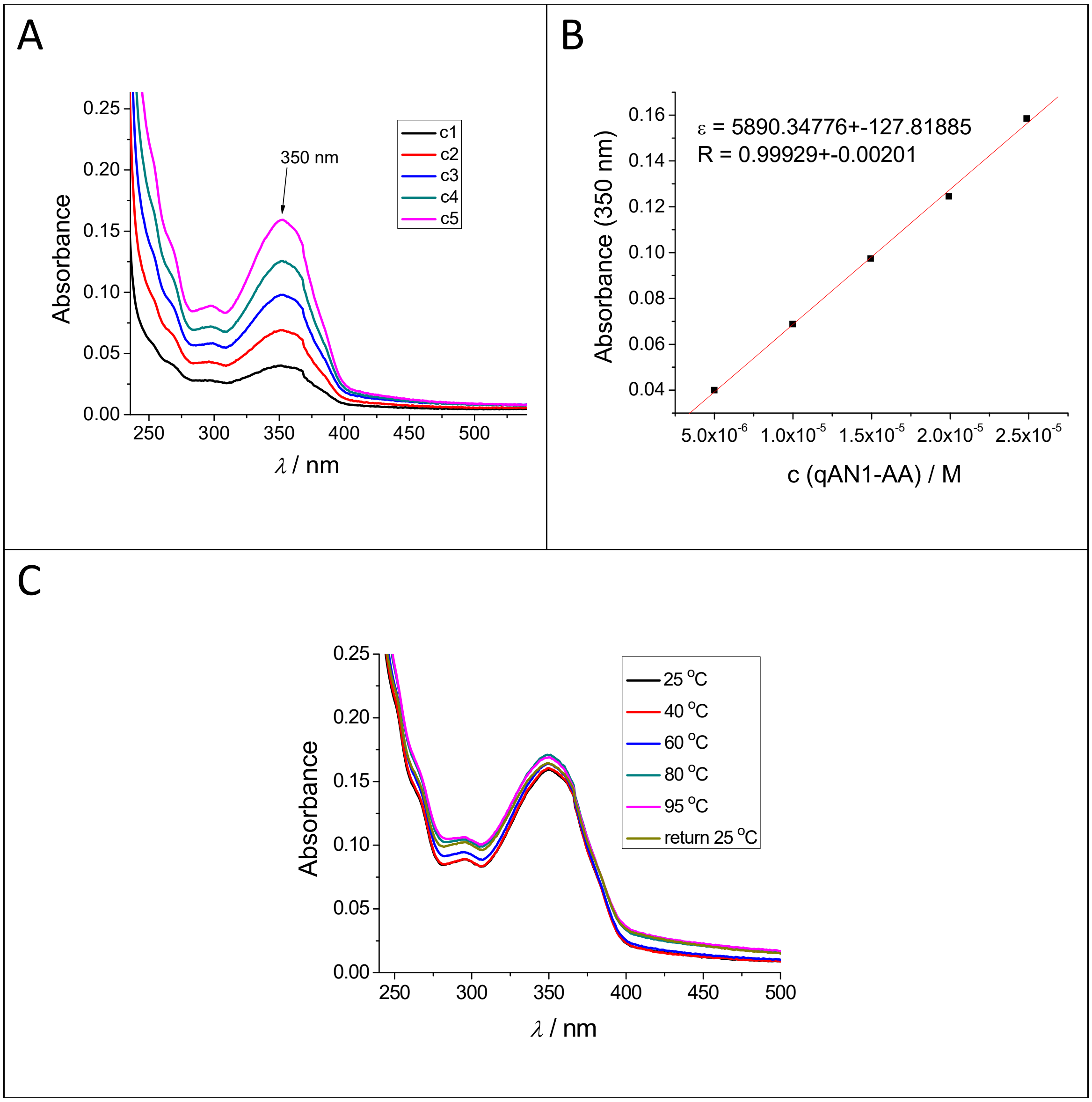

Figure S1. A: UV/Vis spectra of **qAN1-AA**, $c = 5\times10^{-6}$ - 2×10^{-5} M; B: linear dependence (—) of the absorbance at 350 nm (■) on the **qAN1-AA** concentration; C: temperature dependence of UV/Vis spectra ($c = 2\times10^{-5}$ M). Performed in Na-cacodylate buffer, pH = 7.0, $I = 0.05$ M.

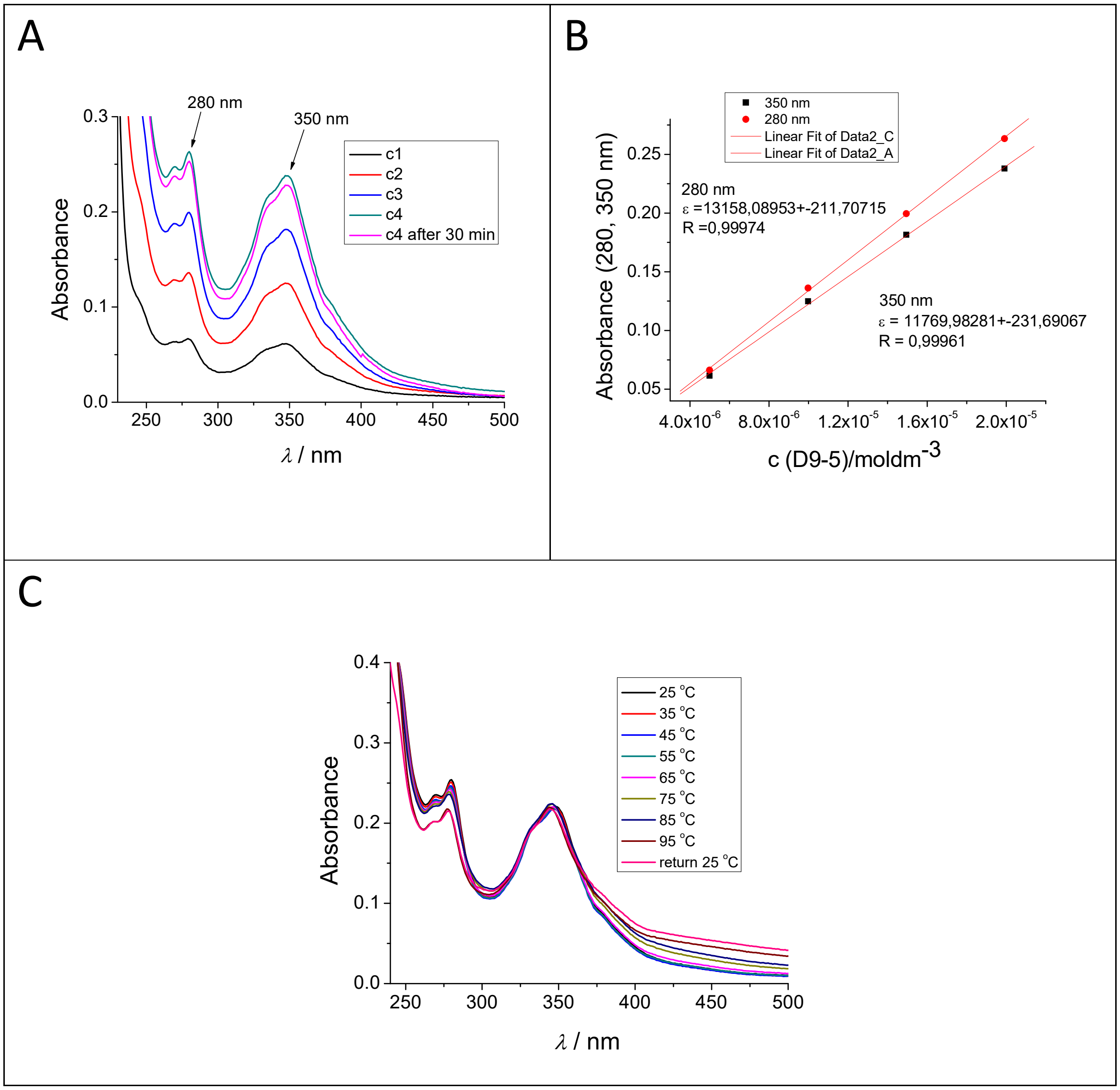

Figure S2. A: UV/Vis spectra of **qAN1-pyr1**, $c = 5\times10^{-6}$ - 2×10^{-5} M; B: linear dependence (—) of the absorbance at 280 (■) and 350 nm (■) on the **qAN1-pyr1** concentration; C: temperature dependence of UV/Vis spectra (c = 2×10^{-5} M). Performed in Na-cacodylate buffer, pH = 7.0, I = 0.05 M.

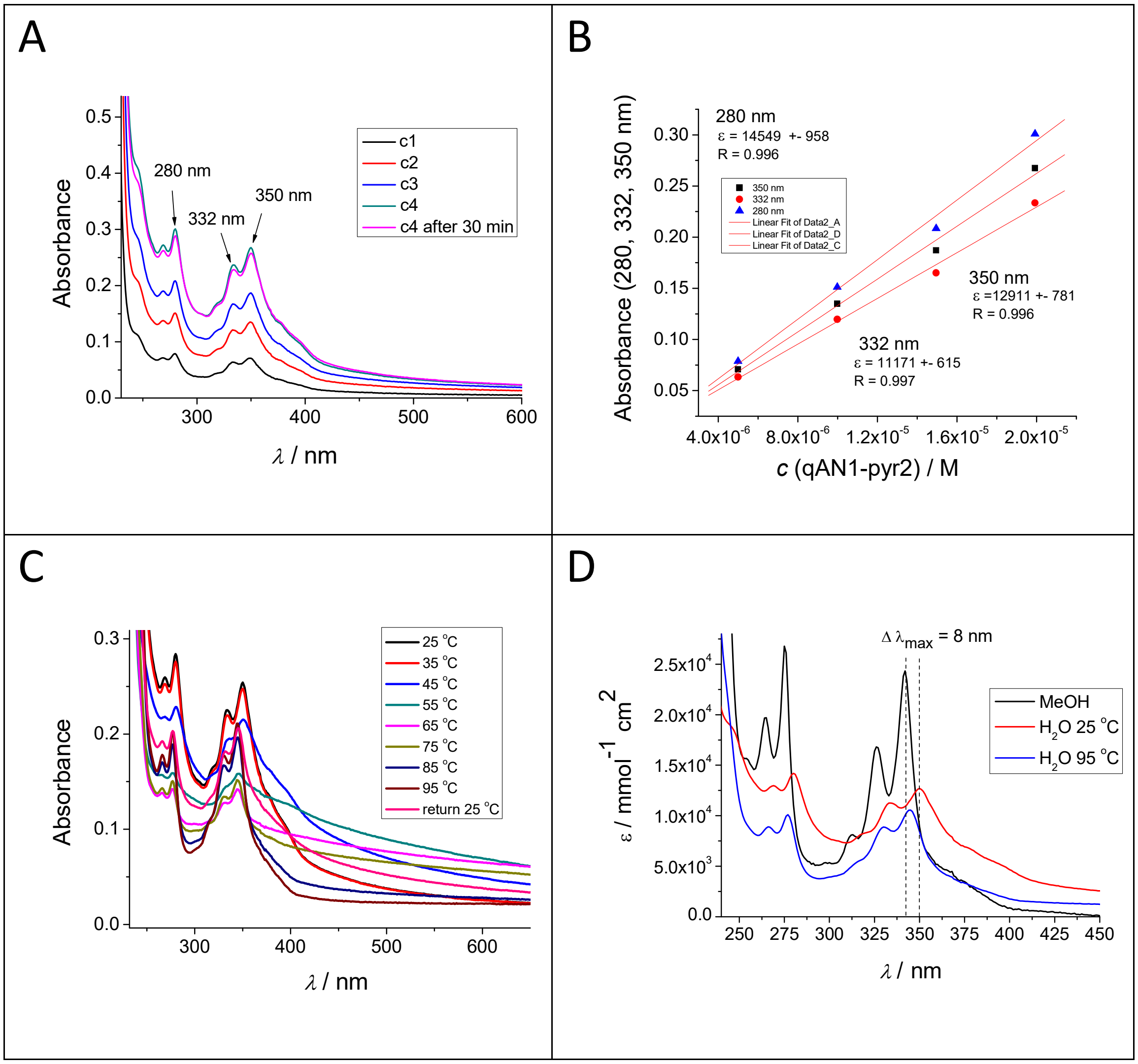

Figure S3. A: UV/Vis spectra of **qAN1-pyr2**, $c = 5\times10^{-6}$ - 2×10^{-5} M; B: linear dependence (—) of the absorbance at 280 (▲), 332 (●) and 350 nm (■) on the **qAN1-pyr2** concentration; C: temperature dependence of UV/Vis spectra ($c = 2\times10^{-5}$ M); D: overlapped spectra in water (25 ºC and 95 ºC) and in methanol (25 ºC). Performed in Na-cacodylate buffer, pH = 7.0, I = 0.05 M.

Table S1. Electronic absorption data of **qAN1-AA**, **qAN1-pyr1** and **qAN1-pyr2** at pH 7, sodium cacodylate buffer, I = 0.05 M.

Compound	λ_{max}/nm	10^3/mmol^{-1} cm^2
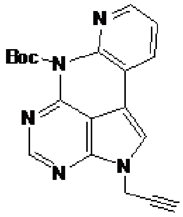 **4**	350	7.9±0.1
**qAN1-AA**	350	5.9±0.1
**qAN1-pyr1**	280<br>350	13.2±0.2<br>11.8±0.2
**qAN1-pyr2**	280<br>332<br>350	14.5±0.1<br>11.2 ±0.1<br>12.9±0.1

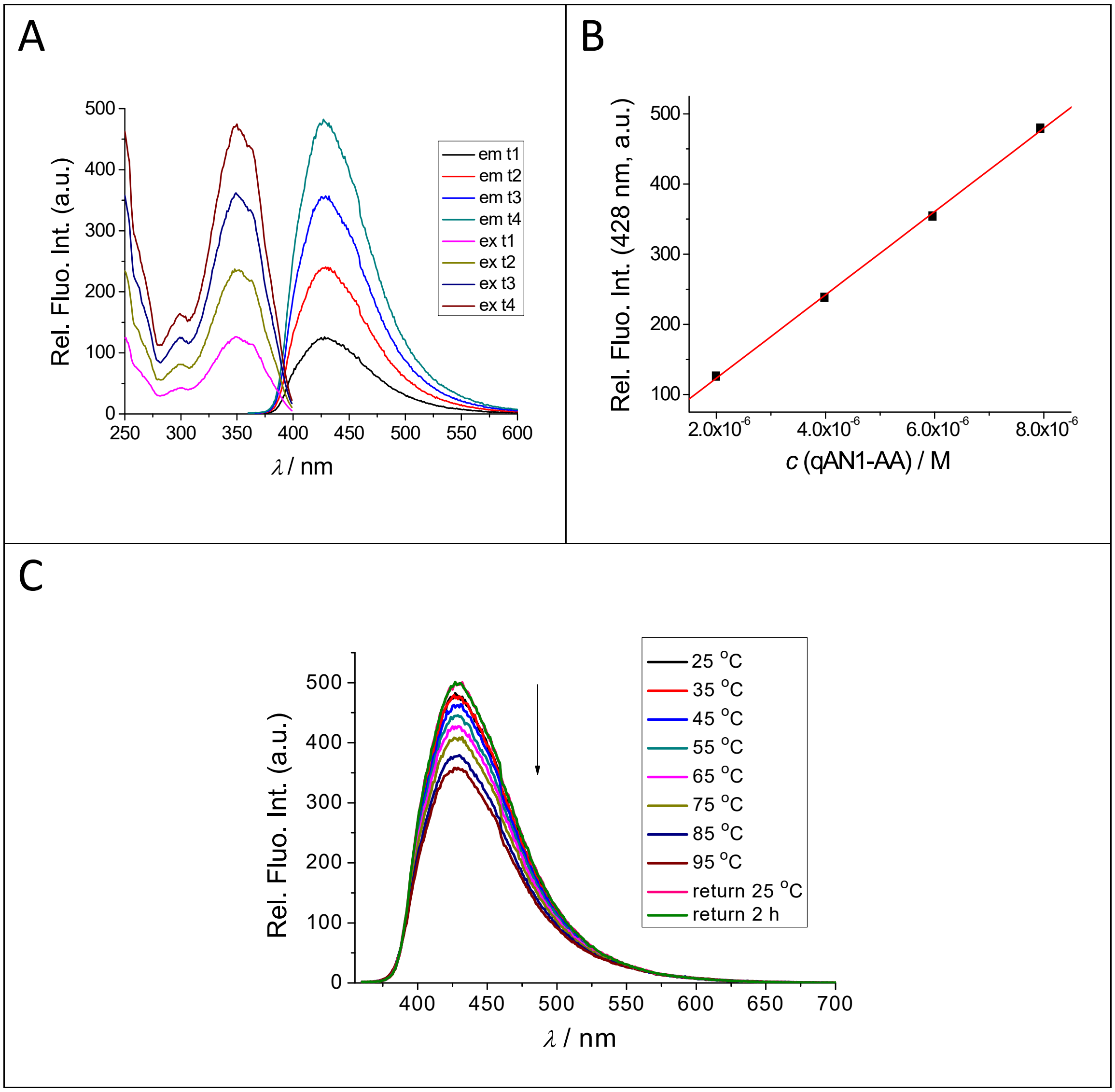

Figure S4. A: Excitation (λ_{em}= 428 nm) and emission spectra of **qAN1-AA** (λ_{exc}= 350 nm); B: dependence of emission (λ_{em}= 428 nm) on *c*(**qAN1-AA**); C: temperature dependence (c = 8×10^{-6} M). Performed in buffer sodium cacodylate, pH 7.0, I = 0.05 M.

Fluorimetric titrations:

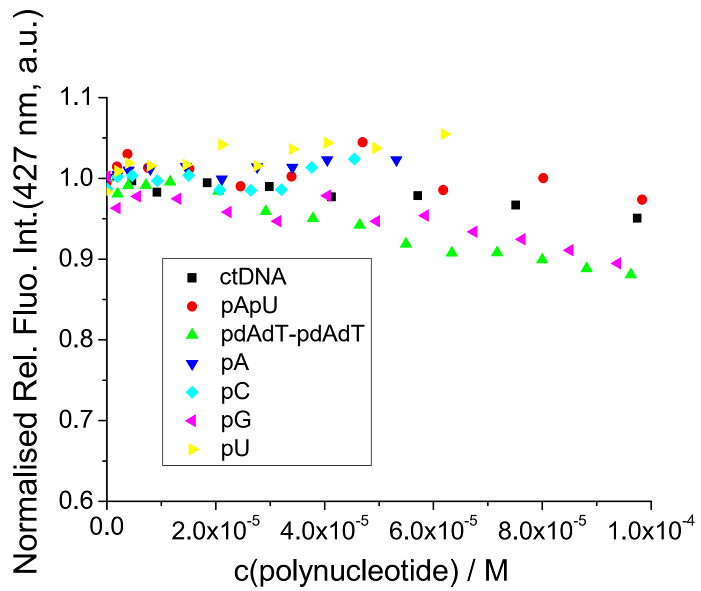

Figure S5. Fluorimetric titration of **qAN1-AA** (c = 1×10^{-6} M; λ_{exc}= 350 nm) with ds-DNA, ds-RNA and ss-RNA, in the range $r_{[\mathbf{qAN1\text{-}AA}]\,/\,[\text{polynucleotide}]}$ = 1-0.01. Performed at pH 7, sodium cacodylate buffer, I = 0.05 M.

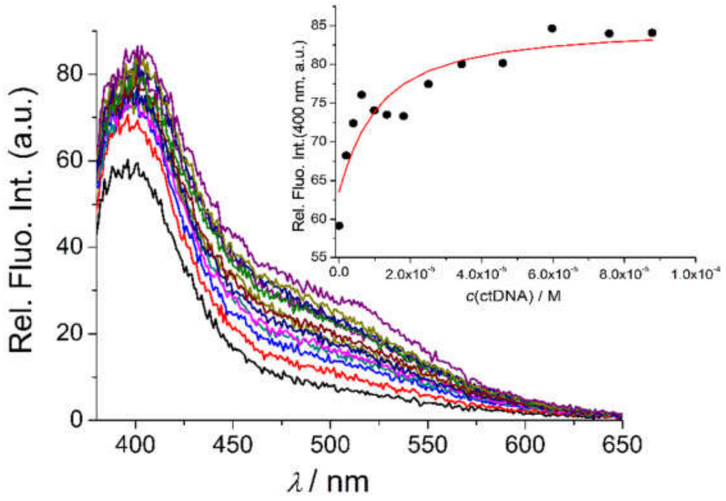

Figure S6. Fluorimetric titration of **qAN1-pyr1** (c = 1×10^{-6} M; λ_{exc}= 350 nm) with ctDNA**.** Performed at pH 7, sodium cacodylate buffer, I = 0.05 M.

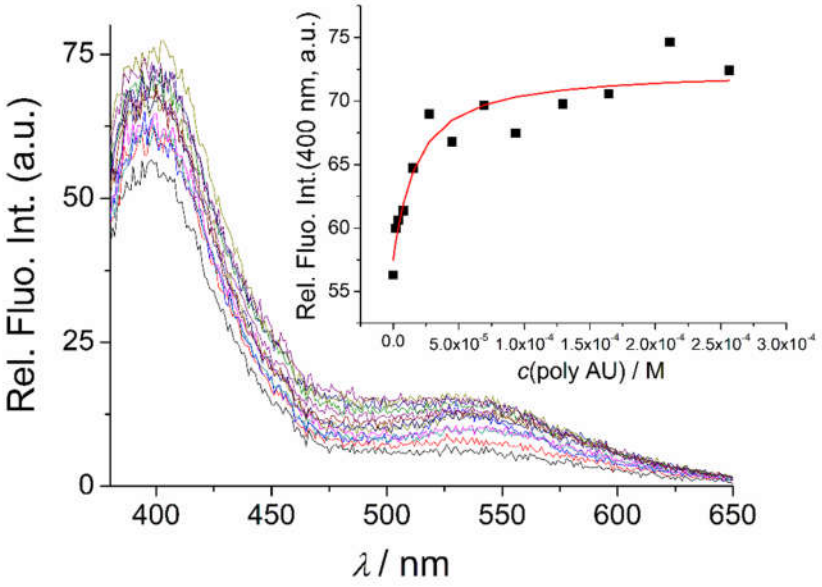

Figure S7. Fluorimetric titration of **qAN1-pyr1** (c = 1×10^{-6} M; λ_{exc}= 350 nm) with poly AU. Performed at pH 7, sodium cacodylate buffer, I = 0.05 M.

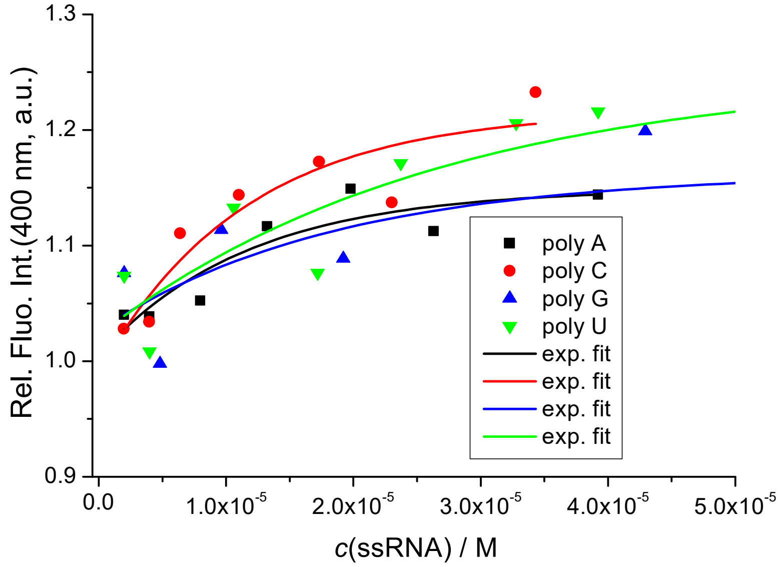

Figure S8. Fluorimetric titration of **qAN1-pyr1** (c = 1×10^{-6} M; λ_{exc}= 350 nm) with ss-RNA, in the range $r_{[\textbf{qAN1-pyr1}] / [ssRNA]}$ = 1-0.01. Performed at pH 7, sodium cacodylate buffer, I = 0.05 M.

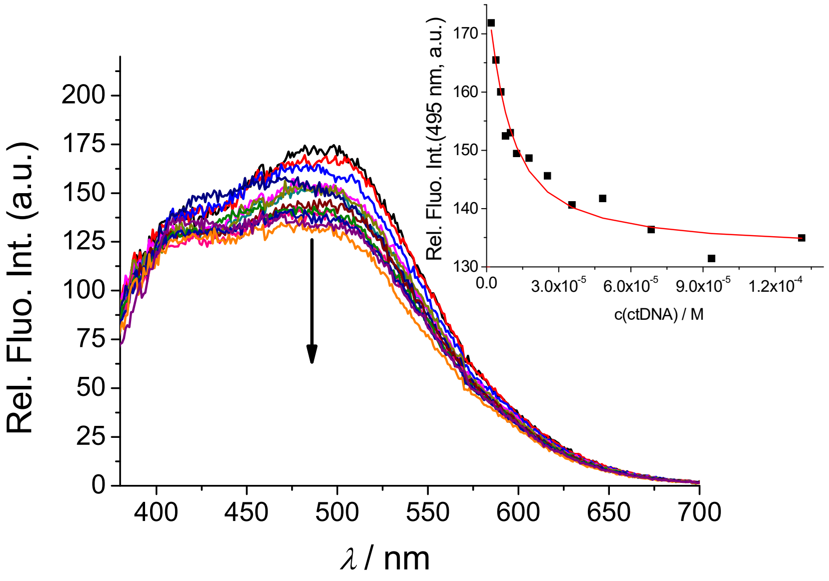

Figure S9. Fluorimetric titration of **qAN1-pyr2** (c = 1×10^{-6} M; λ_{exc}= 350 nm) with ctDNA**.** Performed at pH 7, sodium cacodylate buffer, I = 0.05 M.

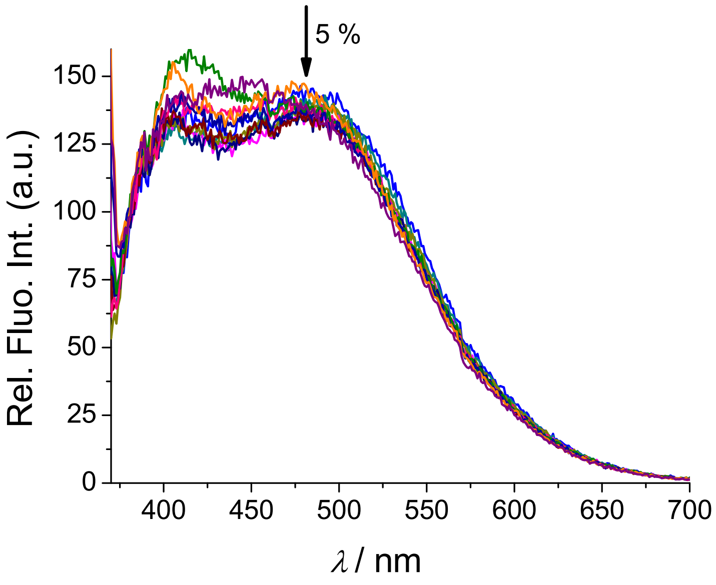

Figure S10. Fluorimetric titration of **qAN1-pyr2** (c = 1×10^{-6} M; λ_{exc}= 350 nm) with poly AU**.** Performed at pH 7, sodium cacodylate buffer, I = 0.05 M.

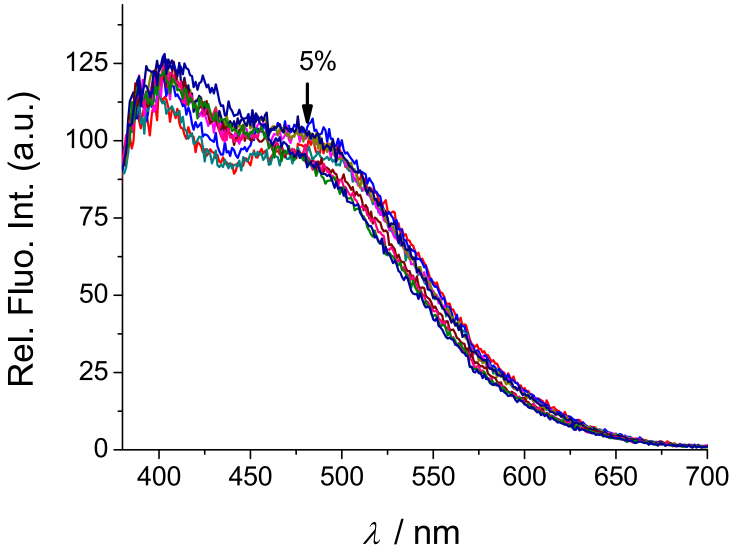

Figure S11. Fluorimetric titration of **qAN1-pyr2** (c = 1×10^{-6} M; λ_{exc}= 350 nm) with poly C**.** Performed at pH 7, sodium cacodylate buffer, I = 0.05 M. Too small changes for accurate fitting of data to Scatchard eq.

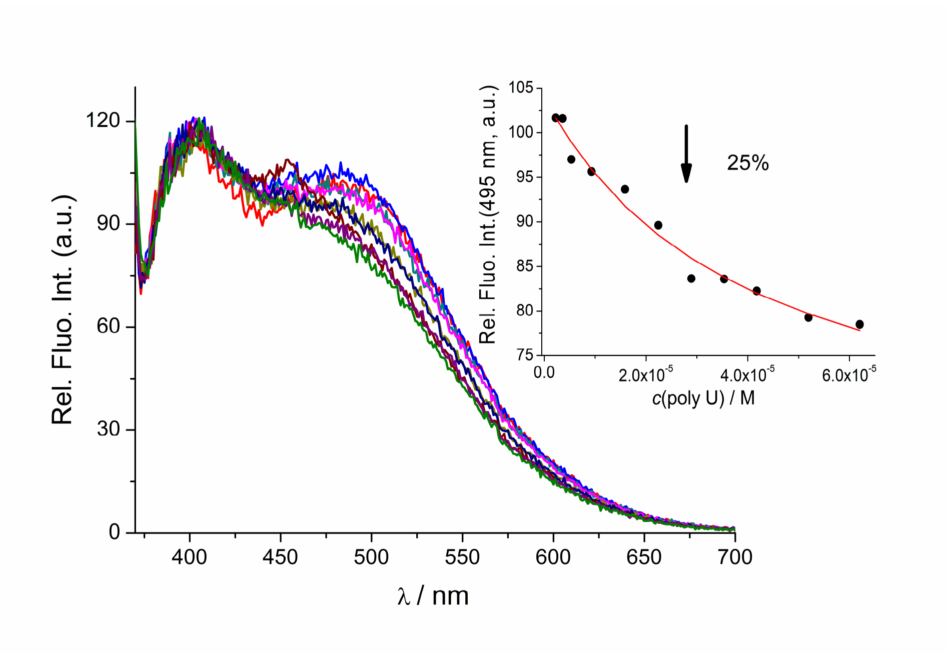

Figure S12. Fluorimetric titration of **qAN1-pyr2** (c = 1×10^{-6} mol dm^{-3}; λ_{exc}= 350 nm) with poly U. Performed at pH 7, sodium cacodylate buffer, I = 0.05 mol dm^{-3}.

Processing of titration data by non-linear fitting to Scatchard eq.

By measuring the spectral changes of the ligand as a function of polynucleotide concentration, binding isotherms can be generated and used to calculate binding constants.

Large errors with Scatchard analysis are often encountered (see I. R. Klotz, Ligand-Receptor Energetics, John Wiley & Sons, Inc. New York, 1997). Since the concentration of observable species can determine the number of binding sites reflected in the isotherm, the apparent stoichiometry can change based upon the concentration of the observable species. In addition, the errors associated with assigning spectral properties of the 100% "free" versus the 100% "bound" become amplified in all the data points, since the fraction bound at each data point is calculated from these two extremes. The data points for the 100% free and the 100% bound states are, therefore, "weighed" much more heavily than the points in the middle of the titration.

On the other hand, non-linear analysis of binding data can help reduce the errors associated with quantifying the spectral properties of these "extreme" (and often inaccurate) data points. Non-linear analysis typically weighs all data points equally and fits all the points to a theoretical curve. However, it is advisable to carefully choose experimental conditions to assure that all dye molecules bind to dominant binding sites – this is done by preliminary experiment for rough estimation of binding affinity and then repeating more detailed titration at conditions of an excess of DNA/RNA binding sites over c(dye), which allows each dye molecule to find its dominant binding site according to J.D. Mc Ghee, P.H. von Hippel formalism for non-cooperative binding (ref. 45 in the manuscript). More detailed considerations how to organize titration experiment and analysis are nicely summarised in J. Lah and G. Vesnaver, J Mol Biol, 2004, 342, 73 (pp 80).

The sensitivity of fluorescence allows titrations to be performed at conditions of large excess of polynucleotide over ligand, which ensures ligand binding in its dominant binding site. As results, both binding stoichiometry and the average affinity of each site are given. Here, data are being processed in Origin programme by Scatchard equation adapted for the non-linear fitting procedure with following parameters and equations (1) and (2):

Parameter Names: K, EpsKompleks, EpsLigand, n

Independent variables: cDNA, cLigand

Dependent variable: A

$$\mathrm{A} = \mathrm{EpsKompleks} * \mathrm{cK} + \mathrm{EpsLigand} * (\mathrm{cLigand} - \mathrm{cK}) + \mathrm{EpsDNA} * (\mathrm{n} * \mathrm{cDNA} - \mathrm{cK}) \quad (1)$$

$$\mathrm{cK} = \left\{ \mathrm{n} * \mathrm{cDNA} + \mathrm{cLigand} + {}^{1}\!/_{\mathrm{K}} - \sqrt{\left[\left(\mathrm{n} * \mathrm{cDNA} + \mathrm{cLigand} + {}^{1}\!/_{\mathrm{K}} \right)^{2} - 4 * \mathrm{n} * \mathrm{cDNA} * \mathrm{cLigand} \right]} \right\} \Big/ 2 \quad (2)$$

Whereby K is binding constant; EpsKompleks is fluorescence intensity of dye/ polynucleotide complex divided by c(dye); EpsLigand is fluorescence intensity of dye divided by c(dye); n = [bound dye] / [polynucleotide]; cDNA is c(polynucleotide); cLigand is c(dye).

Thermal denaturation experiments:

Table S2. The [a]ΔTm values (°C) of ctDNA and poly AU upon addition of ratio [b]***r*** of **qAN1-AA**, **qAN1-pyr1** and **qAN1-pyr2** at pH 7.0 (sodium cacodylate buffer, I = 0.05 mol dm^{-3}).

[a]ΔTm/°C	[b]r = 0.1	[b]r = 0.2	[b]r = 0.3
**qAN1-AA**			
ct-DNA	0	0	0
poly AU	1	1	1
**qAN1-pyr2**			
ct-DNA	0	0	-1
poly AU	0	1	1
**qAN1-pyr1**			
ct-DNA	0	0	- 1
poly AU	1	0.5	0.5

[a] Error in ΔTm : ±0.5°C;
[b] r = [compound] / [polynucleotide];

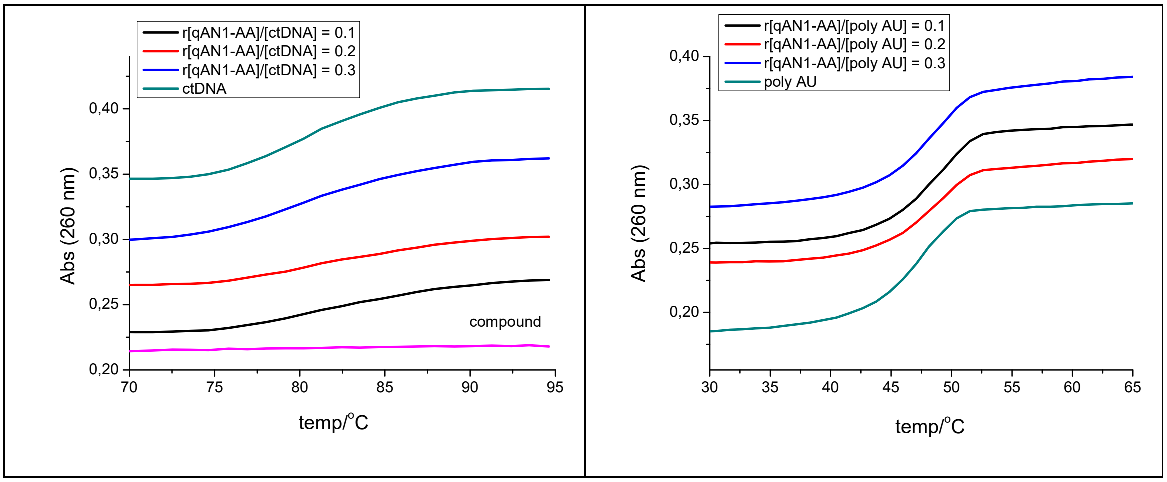

Figure S13. Thermal denaturation curves of **ct-DNA** (c(ct-DNA) = 2.5×10^{-5} M, $r_{[\mathbf{qAN1\text{-}AA}]/[\text{ct-DNA}]}$ = 0.1, 0.2, 0.3) and **poly AU** (c(polyAU) = 2.5×10^{-5} M, $r_{[\mathbf{qAN1\text{-}AA}]/[\text{poly AU}]}$ = 0.1, 0.2, 0.3) at pH 7.0 (sodium cacodylate buffer, I = 0.05 M) upon addition of **qAN1-AA**. Error in ΔTm values: ±0.5 °C.

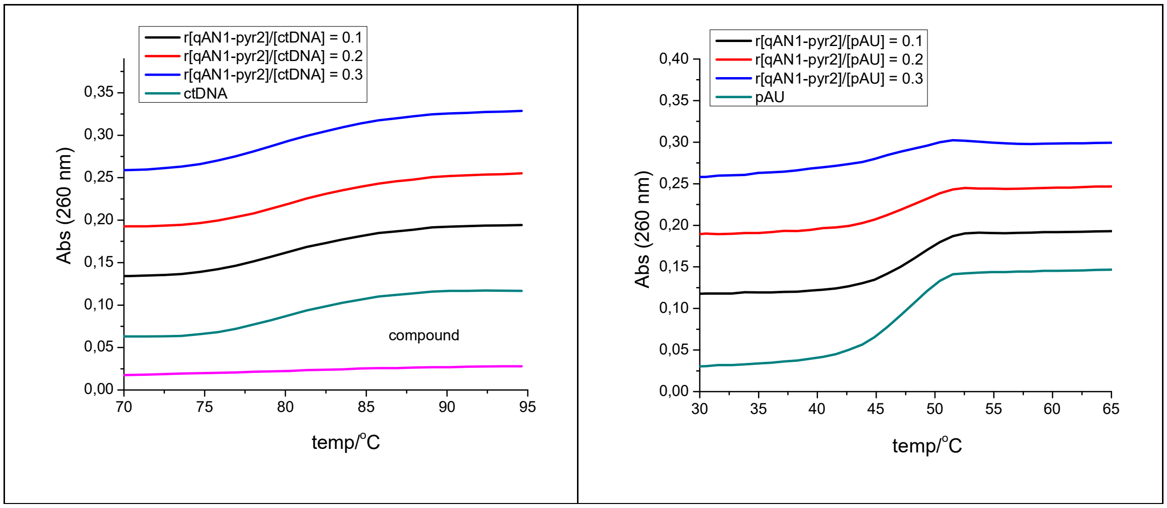

Figure S14. Thermal denaturation curves of **ct-DNA** (c(ct-DNA) = 2.5×10^{-5} M, $r_{[\mathbf{qAN1\text{-}pyr2}]/[\text{ct-DNA}]}$ = 0.1, 0.2, 0.3) and **poly AU** (c(polyAU) = 2.5×10^{-5} M, $r_{[\mathbf{qAN1\text{-}pyr2}]/[\text{poly AU}]}$ = 0.1, 0.2, 0.3) at pH 7.0 (sodium cacodylate buffer, I = 0.05 M) upon addition of **qAN1-pyr2**. Error in ΔTm values: ±0.5 °C.

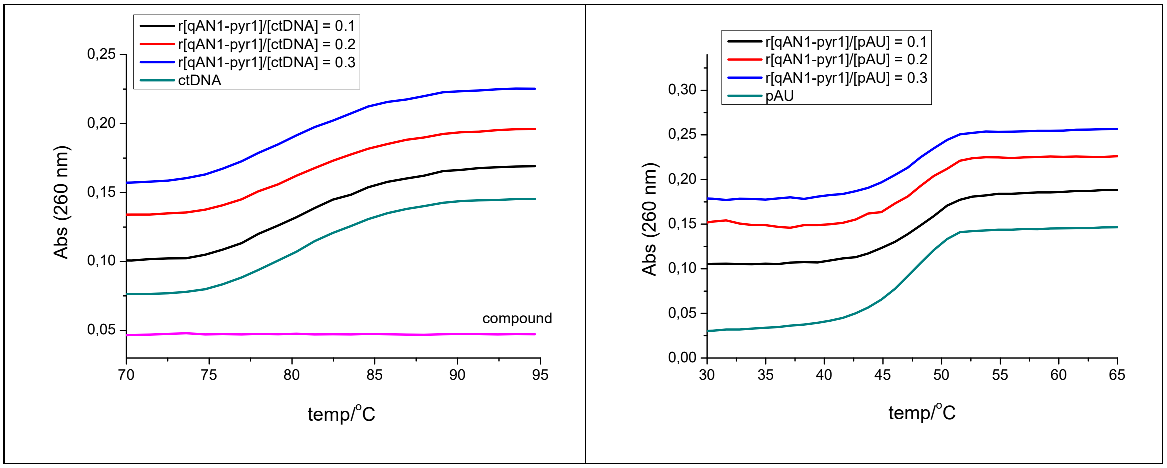

Figure S15. Thermal denaturation curves of **ct-DNA** (c(ct-DNA) = 2.5×10^{-5} M, $r_{[\mathbf{qAN1\text{-}pyr1}]/[\text{ct-DNA}]}$ = 0.1, 0.2, 0.3) and **poly AU** (c(polyAU) = 2.5×10^{-5} M, $r_{[\mathbf{qAN1\text{-}pyr1}]/[\text{poly AU}]}$ = 0.1, 0.2, 0.3) at pH 7.0 (sodium cacodylate buffer, I = 0.05 M) upon addition of **qAN1-pyr1.** Error in ΔTm values: ±0.5 °C.

Circular dichroism:

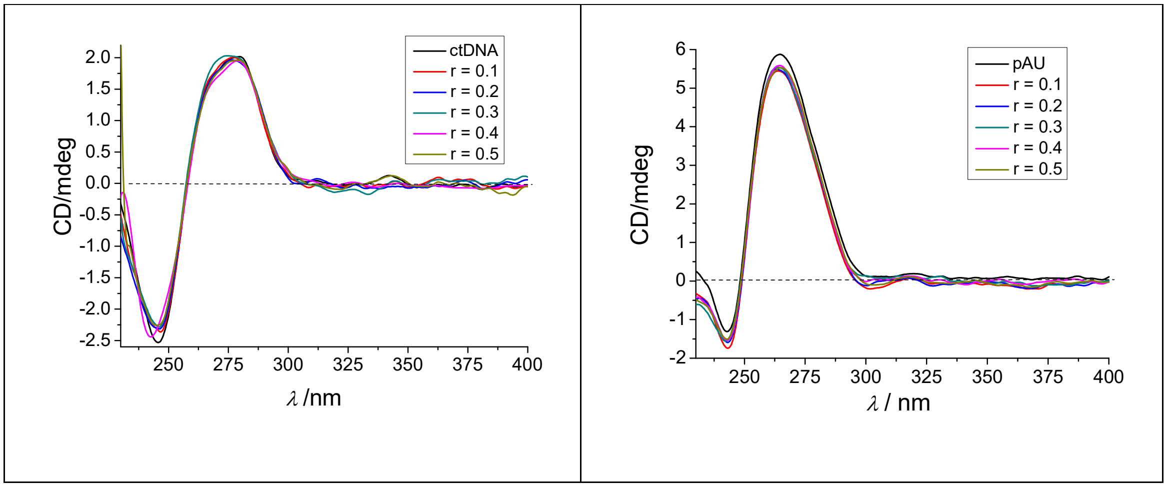

Figure S16. CD titration of **ctDNA** (c=2×10^{-5} M) and **poly AU** (c=2×10^{-5} M) with **qAN1-AA** at molar ratios $\boldsymbol{r}$ = [compound] / [polynucleotide] (pH 7.0, buffer sodium cacodylate, I = 0.05 M).

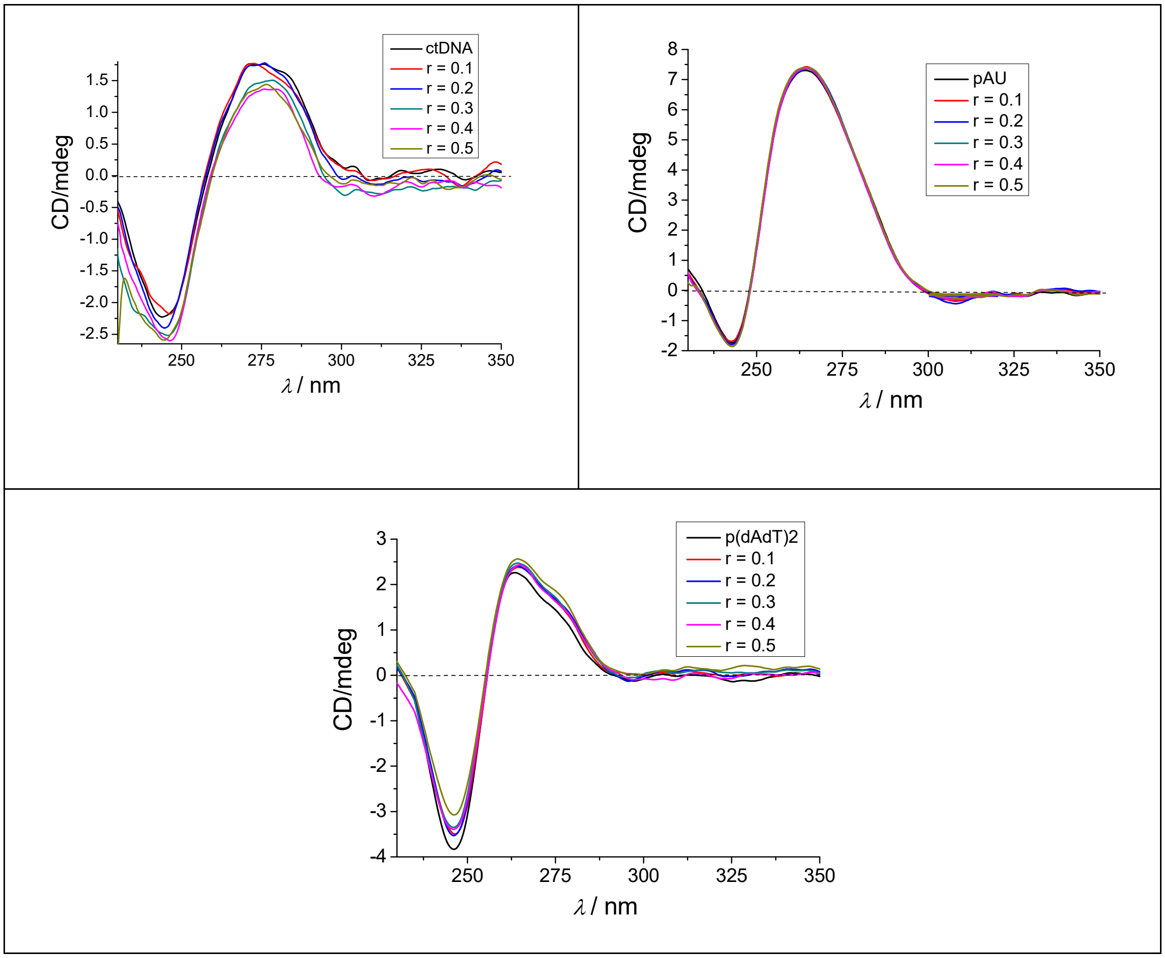

Figure S17. CD titration of **ctDNA** (c=2×10^{-5} M), **poly AU** (c=2×10^{-5} M) and **p(dAdT)$_2$** (c=2×10^{-5} M) with **qAN1-pyr2** at molar ratios $\boldsymbol{r}$ = [compound] / [polynucleotide] (pH 7.0, buffer sodium cacodylate, I = 0.05 M).

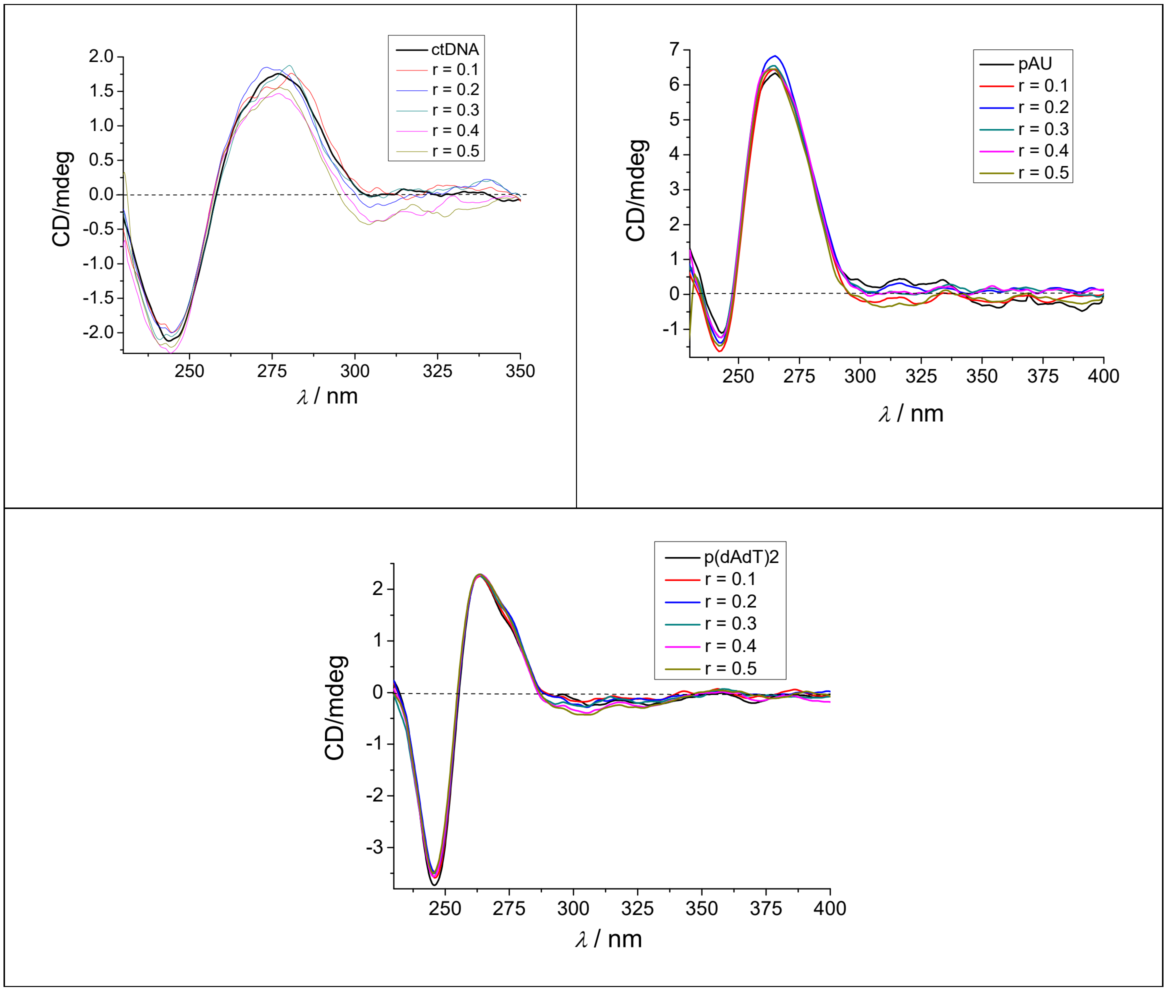

Figure S18. CD titration of **ctDNA** (c=2×10^{-5} M), **poly AU** (c=2×10^{-5} M) and **p(dAdT)$_2$** (c=2×10^{-5} M) with **qAN1-pyr1** at molar ratios ***r*** = [compound] / [polynucleotide] (pH 7.0, buffer sodium cacodylate, I = 0.05 M).

Structural aspects:

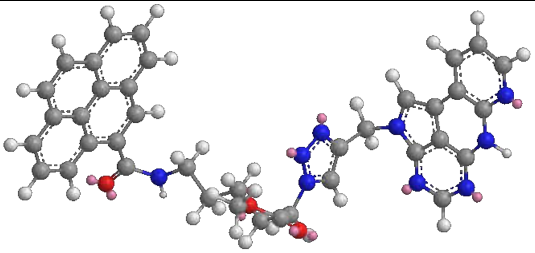

C: qAN1- pyr1

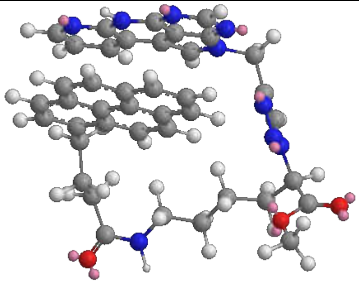

C: qAN1- pyr2

Figure S19. Schematic presentation of intramolecular organization of **qAN1-pyr1** and **qAN1-pyr2** according to UV/vis and fluorimetric data was supported by preliminary energy-minimization done in vacuum: structures were constructed in ChemBio3D program in folded conformation, pyrene and qAN1 being in aromatic stacking distance and then subjected to MM2 energy-minimization (in vacuum). Resulting conformations: qAN1-pyr1 in unfolded confirmation and qAN1-pyr2 remained in self-folded confirmation; are in good agreement with UV/vis and fluorimetric data. However, for accurate structural analysis higher computation level or additional experimental data (e.g. single crystal diffraction or NMR) are needed.

References:

[1] J. B. Chaires, N. Dattagupta, D. M. Crothers, *Biochemistry* **1982**, *21*, 3933.

[2] W. Saenger, *Principles of Nucleic Acid Structure*, Springer-Verlag: New York, **1983**, 226.

[3] C. R. Cantor, P. R. Schimmel, *Biophysical Chemistry,* **1980**, *3*, 1109-1181.
